# Single-photon oxidation of C_60_ by self-sensitized singlet oxygen

**DOI:** 10.1038/s42004-020-0318-x

**Published:** 2020-06-04

**Authors:** Linqi Zhang, Chong Wang, Jiming Bao, A. Kaan Kalkan

**Affiliations:** 1 Functional Nanomaterials Laboratory, Oklahoma State University, Stillwater, OK 74078 USA; 2 Department of Electrical & Computer Engineering, University of Houston, Houston, TX 77204 USA; 3 Present Address: School of Materials and Energy, Yunnan University, Kunming, 650500 P.R. China

**Keywords:** Carbon nanotubes and fullerenes, Photochemistry, Photocatalysis

## Abstract

C_60_ is regarded as the most efficient singlet oxygen (^1^O_2_) photosensitizer. Yet, its oxidation by self-sensitized ^1^O_2_ remains unclear. The literature hints both oxygen and C_60_ must be at excited states to react, implying a two-photon process: first, oxygen is photosensitized (^1^C_60_•^1^O_2_); second, C_60_ is photoexcited (^1^${\mathrm{C}}_{60}^{\ast}$•^1^O_2_). However, this scheme is not plausible in a solvent, which would quench ^1^O_2_ rapidly before the second photon is absorbed. Here, we uncover a single-photon oxidation mechanism via self-sensitized ^1^O_2_ in solvents above an excitation energy of 3.7 eV. Using excitation spectroscopies and kinetics analysis, we deduce photoexcitation of a higher energy transient, ^3^${\mathrm{C}}_{60}^{{\ast}{\ast}}$•^3^O_2_, converting to ^1^${\mathrm{C}}_{60}^{\ast}$•^1^O_2_. Such triplet-triplet annihilation, yielding two simultaneously-excited singlets, is unique. Additionally, rate constants derived from this study allow us to predict a C_60_ half-life of about a minute in the atmosphere, possibly explaining the scarceness of C_60_ in the environment.

## Abstract (web summary)

C_60_ is a highly efficient singlet oxygen (^1^O_2_) photosensitizer, but its oxidation by self-sensitized ^1^O_2_ has not been reported. Here, the authors uncover a single-photon oxidation mechanism via self-sensitized ^1^O_2_ in solvents above an excitation energy of 3.7 eV.

## Introduction

Buckminsterfullerene (C_60_), the most abundant fullerene as well as the most symmetric molecule in nature, has been studied extensively since its discovery in 1985^1^. Among the outstanding properties of C_60_ are ability to accept up to six electrons, high intersystem crossing (ISC) quantum yield, and long-lived triplet states^2^, which have stimulated a thriving research effort for applications in photovoltaics^3^, photocatalysis^4^, and molecular probes^5^. Additionally, the latter two attributes make C_60_ an efficient singlet oxygen (^1^O_2_) sensitizer, particularly promising for photodynamic therapy and environmental remediation^6–8^.

However, C_60_ is subject to photodegradation in these applications at ambient temperature (while thermal oxidation of C_60_ occurs at 370 K and above)^9,10^. The initial work on photooxidation (PO) of C_60_ credited it to ozonation^11–13^, which however is limited to excitation wavelengths shorter than 240 nm for photogeneration of O_3_^14^. Later, however, another reactive oxygen species became the suspect, ^1^O_2_^15–17^. To this end, the most seminal findings have been: (i) unless photoexcited, C_60_ does not react with externally-produced ^1^O_2_^16^; (ii) PO can occur under UV excitation (308 nm) in O_2_ ambient (albeit formation of ^1^O_2_ was not corroborated)^17^; and (iii) C_60_O is the major photoproduct^16–18^. Thus, the PO reaction is anticipated as ${\mathrm{C}}_{60}^{\ast} + {\,}^{1}{\mathrm{O}}_{2} \to {\mathrm{C}}_{60}{\mathrm{O}} + \frac{1}{2}{\mathrm{O}}_{2}$, where both C_60_ and O_2_ must be photoexcited. However, the mechanistic details of the photophysics and photochemistry remain unelucidated. A historical review of the C_60_ PO literature is provided in Supplementary Note 1.

C_60_ has long been known to be an efficient ^1^O_2_ sensitizer. Yet, no evidence has been shown that ^1^O_2_ reacts with its original C_60_ sensitizer, which we refer to as “oxidation with self-sensitized ^1^O_2_”. Here, we present experimental evidence for this phenomenon. Although the lifetime of ^1^O_2_, *τ*, in air is exceptionally long (i.e., 45 min)^19^, it shortens to microseconds to nanoseconds in solvents. Inspired by this broad range of *τ*, we investigated PO of C_60_ in hexane (C_6_H_14_), chloroform (CHCl_3_), and carbon tetrachloride (CCl_4_), where *τ* is 30, 207 and 87,000 μs, respectively^20^. Our kinetics study reveals C_60_ concentration decays exponentially under UV excitation and the decay rate increases with *τ*. We also show the decay dominantly occurs as a single-photon process above the photon energy (*h*υ) threshold of 3.7 eV, being the onset of 1^1^A_g_ → 2^1^H_u_ transition in C_60_.

## Results and discussion

### Absorption spectroscopy

**Fig. 1:**
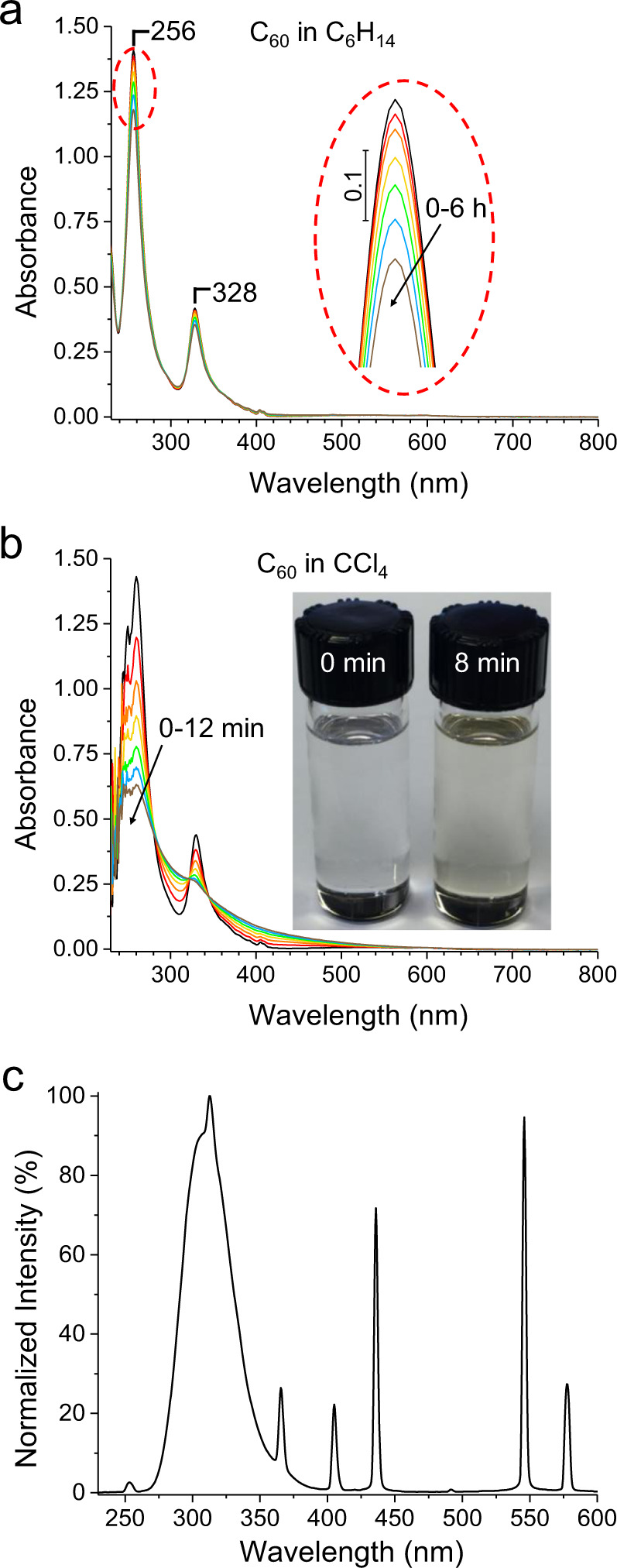
Time series absorbance spectra of C_60_ under UV excitation of 3.74 mW/cm^2^. **a** In C_6_H_14_; **b** in CCl_4_. The inset of (**b**) shows photos of C_60_ solutions, unexposed and after 8 min of UV exposure. **c** The spectrum of the excitation source, UVP XX-15 UV lamp.

Figure 1a, b shows time series optical absorption spectra for our slowest and fastest PO kinetics, which occur in C_6_H_14_ and CCl_4_, respectively. The major C_60_ absorption peaks at 256 and 328 nm are seen to decrease systematically, while the baseline rises indicative of a photoproduct, which is also evident from yellowing of the solution (Fig. 1b, inset). The spectrum of the excitation source (Fig. 1c) consists of a major narrow band peaking at 310 nm with no emissions below 250 nm. Hence, the possibility of O_3_ generation is ruled out^14^. In Fig. 1b, the noise below 255 nm is due to the high absorption of CCl_4_ attenuating the optical beam. However, it does not deteriorate the accuracy of the absorption peak of C_60_ at 260 nm in CCl_4_ (see Supplementary Discussion).

### Phosphorescence spectroscopy

**Fig. 2:**
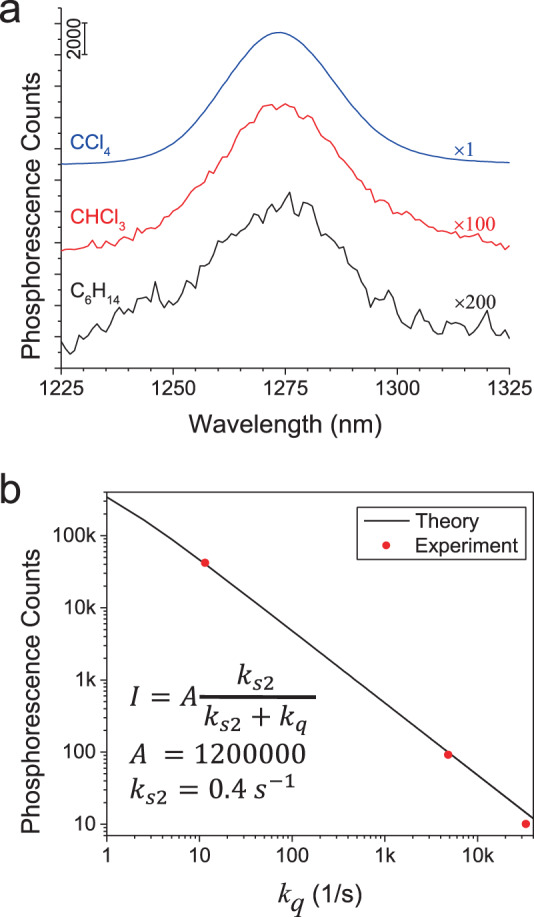
Phosphorescence of ^1^O_2_ sensitized by C_60_. **a** Spectra in different solvents (under 375 nm radiation of 9.5 mW/cm^2^ intensity). Here, [C_60_] in C_6_H_14_ is 3.92 times higher than the usual concentration. **b** Match of theoretical intensity (*I*) with experiment (in (**a**)) validating the *k*_*q*_ values adopted from the literature. *k*_*s*2_ (0.4 s^−1^) is the sensitization rate by C_60_. The 2 in the subscript indicates $h\upsilon$ < 3.7 eV (Supplementary Results).

In Fig. 2a, the phosphorescence peak at 1273 nm substantiates photosensitization of ^1^O_2_ by C_60_ in the three solvents in our study. Figure 2b plots the measured phosphorescence peak intensity versus quenching rate constant (*k*_*q*_ = 1/*τ*). Here, values of *τ* for the three solvents are borrowed from ref. ^20^ as listed above. The experimental data points match with the theoretical trend (Supplementary Eq. (48)). Hence, we confidently adopt the *τ* values from the literature.

### Vibrational spectroscopy

**Fig. 3:**
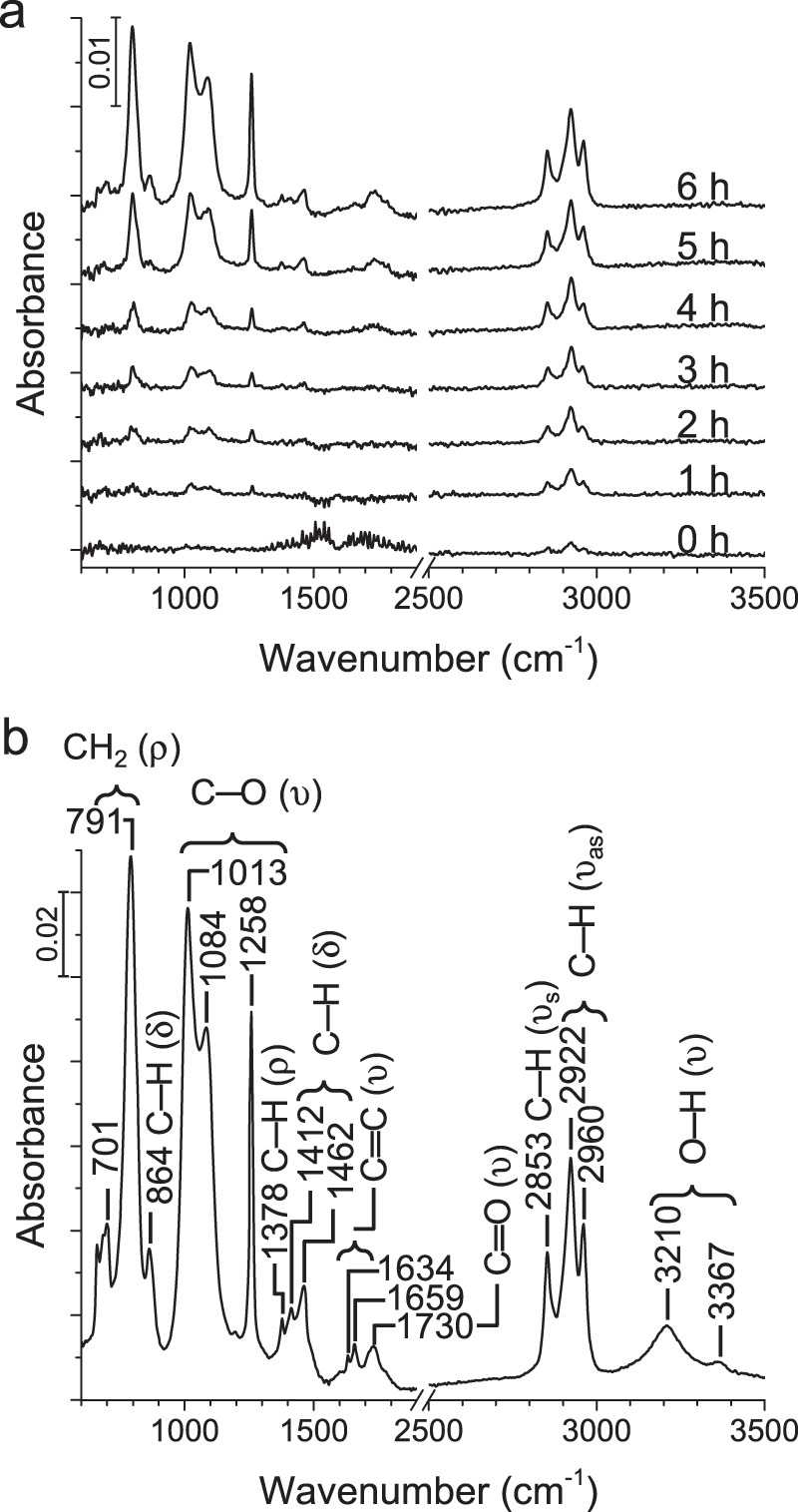
Time series FTIR spectra. **a** C_60_ in CHCl_3_ under the same UV exposure conditions as in Fig. 1. **b** Assignment of FTIR peaks after 6 h of UV exposure (ρ: rocking; δ: bending; υ: stretching).

PO of C_60_ is also characterized by the Fourier-transform infrared (FTIR) spectra in Fig. 3a, where C–O, C═O, and O–H stretching vibrations are indicative of C_60_ oxidation^13^. We anticipate the O–H groups result from the Norrish type II reaction^21^. The evolution of C–H vibrational peaks suggest fragmentation of the C_60_ cage subsequent to PO. The peak frequencies and important assignments are shown in Fig. 3b^22,23^. Detailed peak assignments are given in Supplementary Table 1.

### Mechanism of C_60_ photooxidation in solvents

**Fig. 4:**
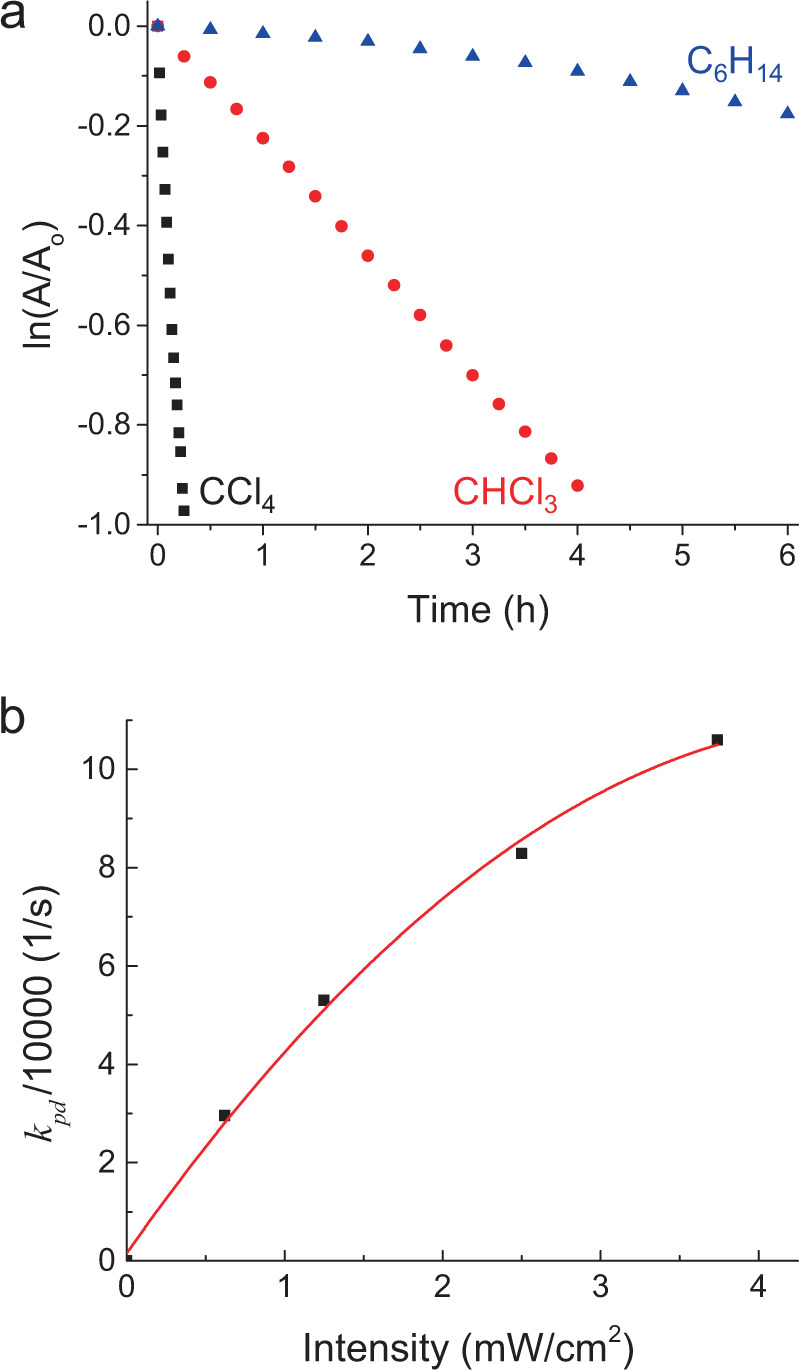
C_60_ oxidation kinetics. **a** C_60_ time decay in different solvents. *A* is optical absorbance at 256 nm (peak), *A*_o_ being the original value (prior to exposure). Although the photoproduct baseline is not subtracted, the slopes represent exponential decay rates (*k*_pd_) with minimal error as corroborated in Supplementary Discussion. The UV exposures are same as in Fig. 1. **b** *k*_pd_ in CCl_4_ as a function of irradiation intensity.

At first, we are inclined to explain the oxidation of C_60_ by its reaction with free ^1^O_2_. In this model, ^1^O_2_ is released to the solvent after photosensitization by a C_60_. Subsequently, it collides and reacts with a C_60_ unless quenched by the solvent. This straightforward model (detailed in Supplementary Information; Kinetics Model) is consistent with our observation that PO rate increases with *τ*. Additionally, its rate is quadratic in [C_60_] as well as radiation intensity. On the contrary, the kinetics of C_60_, as monitored from optical absorption (Fig. 4a) suggests exponential decay, i.e., $\frac{\mathrm{d}}{{\mathrm{d}t}}\left[ {\mathrm{C}}_{60} \right] = - k_{\mathrm{pd}}\left[ {\mathrm{C}}_{60} \right]$, where *k*_pd_ is the C_60_ photodecay rate. Additionally, Fig. 4b establishes a linear dependence of *k*_pd_ on excitation intensity, and hence a single-photon process. Thus, we rule out “oxidation with free ^1^O_2_” as the dominant PO mechanism. Instead, consistent with the observed exponential decay, we propose “oxidation with self-sensitized ^1^O_2_”, where a C_60_ molecule photosensitizes a ^1^O_2_ and reacts with that same ^1^O_2_ in a collision complex:

1 $$	{\mathrm{C}}_{60} + {\,}^{3}{\mathrm{O}}_{2}\mathop{\longrightarrow}\limits^{{\mathrm{collision}}}{\mathrm{C}}_{60} \bullet {\,}^{3}{\mathrm{O}}_{2}\mathop{\longrightarrow}\limits^{{hv}}{\mathrm{C}}_{60}^ \ast \bullet {\,}^{3}{\mathrm{O}}_{2}\mathop{\longrightarrow}\limits^{{\mathrm{sensitization}}}\\ 	\hskip 50pt {\mathrm{C}}_{60}\bullet {\,}^{1}{\mathrm{O}}_{2}\mathop{\longrightarrow}\limits^{\mathrm{oxidation(?)}}{\mathrm{C}}_{60}{\mathrm{O}}$$

Yet, Scheme (1) has a flaw with the oxidation step, where C_60_ and ^1^O_2_ will not react, because C_60_ must be excited to ${\mathrm{C}}_{60}^{\ast}$. To meet this condition, a two-photon process could be proposed, where the first photon excites C_60_ to sensitize ^1^O_2_ and the second one excites C_60_ to a high energy singlet state, which thereafter reacts with ^1^O_2_:

2 $$	{\mathrm{C}}_{60} + {\,}^{3}{\mathrm{O}}_{2}\mathop{\longrightarrow}\limits^{{\mathrm{collision}}}{\mathrm{C}}_{60} \bullet {\,}^{3}{\mathrm{O}}_{2}\mathop{\longrightarrow}\limits^{{h\upsilon }}{\mathrm{C}}_{60}^ \ast \bullet {\,}^{3}{\mathrm{O}}_{2}\mathop{\longrightarrow}\limits^{{\mathrm{sensitization}}}\\ 	\hskip 25pt {\mathrm{C}}_{60}\bullet {\,}^1{\mathrm{O}}_{2}\mathop{\longrightarrow}\limits^{{h\upsilon }}{\mathrm{C}}_{60}^ \ast \bullet {\,}^1{\mathrm{O}}_{2}\mathop{\longrightarrow}\limits^{{\mathrm{oxidation}}}{\mathrm{C}}_{60}\mathrm{O}$$

However, a two-photon process is already excluded by our results (i.e., Fig. 4b). Additionally, Scheme (2) can be ruled out by fundamental considerations. Even the longest *τ* (0.087 s in CCl_4_) is significantly shorter than the period between two subsequent excitations of C_60_, being 3.7 s (see Supplementary Information; Kinetics Model). Therefore, before the second photon absorption occurs in Scheme (2), C_60_•^1^O_2_ will relax to C_60_•^3^O_2_ with a high probability. In other words, the excited O_2_ and excited singlet C_60_ will hardly coincide in time.

Accordingly, we are urged to consider a scheme, which allows simultaneous excitation of O_2_ and C_60_, after which they coexist and react. Scheme (1) considers the most basic photosensitization event, where C_60_ returns to its ground singlet state after imparting its energy to O_2_. On the other hand, it is possible that C_60_ returns to an excited singlet state, ${\mathrm{C}}_{60}^{\ast}$, (if it is photoexcited to a sufficiently high energy singlet state, ${\mathrm{C}}_{60}^{{\ast}{\ast}}$). Accordingly, Scheme (1) may be modified to:

3 $$	{\mathrm{C}}_{60} + {\,}^{3}{\mathrm{O}}_{2}\mathop{\longrightarrow}\limits^{{\mathrm{collision}}}{\mathrm{C}}_{60} \bullet {\,}^{3}{\mathrm{O}}_{2}\mathop{\longrightarrow}\limits^{{h\upsilon }}{\mathrm{C}}_{60}^{ \ast \ast } \bullet {\,}^{3}{\mathrm{O}}_{2}\mathop{\longrightarrow}\limits^{{\mathrm{sensitization}}}\\ 	\hskip 50pt {\mathrm{C}}_{60}^{\ast}\bullet {\,}^{1}{\mathrm{O}}_{2}\mathop{\longrightarrow}\limits^{{\mathrm{oxidation}}}{\mathrm{C}}_{60}{\mathrm{O}}$$

The lowest energy ^1^${\mathrm{C}}_{60}^{\ast}$ is 2.33 eV above the ground state. Additionally, ^1^O_2_ sensitization requires 0.98 eV while 0.37 eV is lost to exchange interaction during singlet-to-triplet conversion^24^. Therefore, a minimum excitation energy (*h*υ) of 3.68 eV is needed for Scheme (3) (Supplementary Fig. 4) to succeed. Consistently, our investigation using 455 and 395 nm LED excitations (2.73 and 3.14 eV) with similar photon-count exposures as in Fig. 1 yielded no detectable PO, although we confirmed ^1^O_2_ sensitization for these excitations from the 1273 nm phosphorescence (Supplementary Fig. 1). In the below two paragraphs, we experimentally corroborate 1^1^A_g_ → 2^1^H_u_ is the major driver of C_60_ PO in the solvents. Interestingly, 1^1^A_g_ → 2^1^H_u_ starts at 3.72 eV^25^, being very close to the threshold energy for PO (Scheme (3)).

**Fig. 5:**
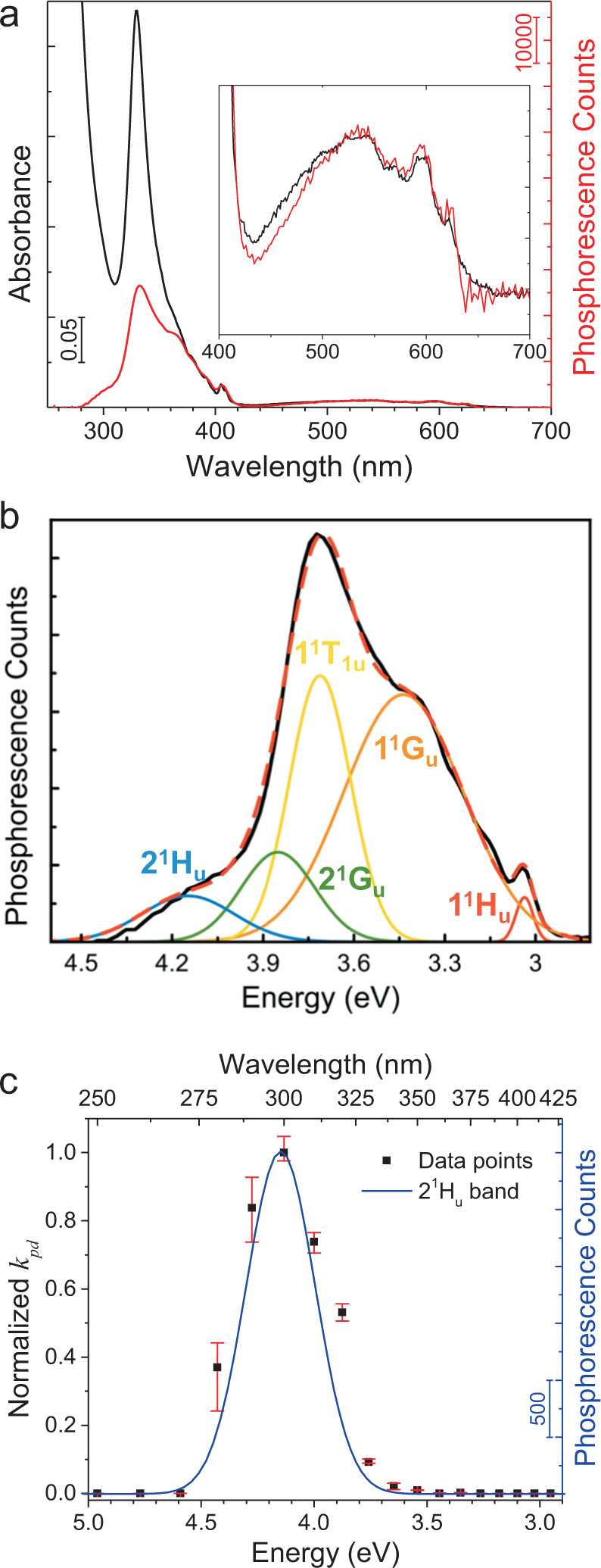
Excitation spectra. **a** Overlay of the absorption spectrum of C_60_ (black) and excitation spectrum for photosensitization of ^1^O_2_ by C_60_, monitored from ^1^O_2_ phosphorescence counts at 1270 nm (red). **b** Deconvolution of the phosphorescence excitation spectrum. **c** Overlay of normalized *k*_pd_ at different excitation wavelengths (black) and the deconvoluted 2^1^H_u_ band (blue). Confidence interval error bars are shown (red) after 3 independent measurements.

In Fig. 5a, excitation spectrum for ^1^O_2_ phosphorescence (sensitization) closely follows C_60_ absorption spectrum from 700 nm down to 370 nm. This trend is consistent with constant and near-unity ^1^O_2_ photosensitization quantum yield by C_60_, Φ_*s*_, as established in the literature^24^. Figure 5b shows the deconvolution of the excitation spectrum to Gaussians below 400 nm. Each band marks an optical transition. Although these transitions may also be resolved from optical absorption, their deconvolution is more facile from our excitation spectrum.

Below 370 nm, however, Φ_*s*_ diverges from the absorption spectrum and drops significantly. On the other hand, the excitation spectrum for oxidation (i.e., *k*_pd_) in Fig. 5c (“Methods”) exhibits a reverse trend. The PO rate, *k*_p__d_, is essentially zero for the spectral range, where Φ_*s*_ is at its maximum value of unity, but it is activated at the threshold of about 335 nm (3.70 eV), at which Φ_*s*_ is reduced to 0.37. Hence, the excitation trends in Fig. 5a, c, being spectrally different, underscore the fact that sensitization of ^1^O_2_ is not sufficient for the oxidation of C_60_. Specifically, as seen in Fig. 5c, the *k*_pd_ spectrum matches the 2^1^H_u_ band. These findings validate Scheme (3) as well as 1^1^A_g_ → 2^1^H_u_ being the major driver of PO. Here, the normalized *k*_pd_ values were derived from the ^1^O_2_ phosphorescence intensity (i.e., counts proportional to [C_60_]) kinetics (Supplementary Fig. 3).

**Fig. 6:**
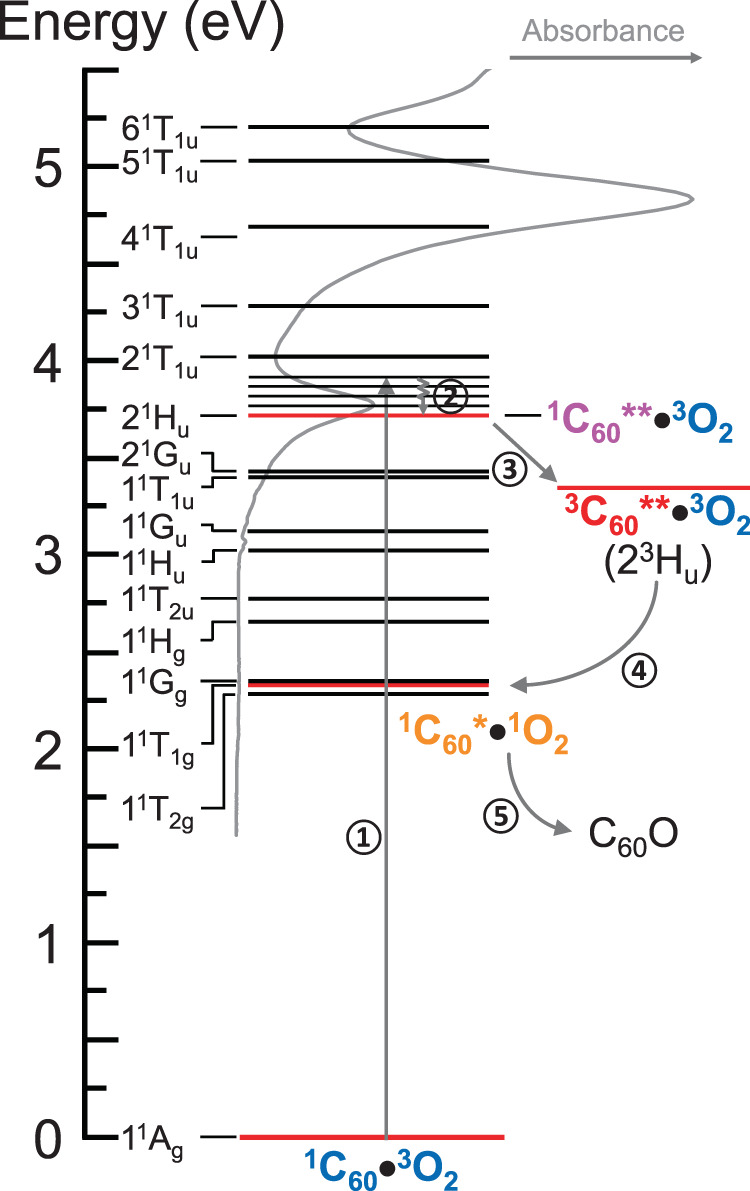
Jablonski diagram illustrating photooxidation of C_60_. To a first approximation, we adopt the energy structure of isolated C_60_ for the C_60_ of C_60_•O_2_. The gray curve represents the absorbance spectrum of C_60_.

While 1^1^A_g_ → 2^1^H_u_ is the major driver of C_60_ PO (Scheme (3)), 1^1^G_u_, 1^1^T_1u_, and 2^1^G_u_ states can also be excited to their vibronic levels higher than 3.7 eV (from 1^1^A_g_), as inferred from their deconvoluted bands in Fig. 5b. However, vibrational relaxation (VR) is the fastest process, quickly quenching 1^1^G_u_, 1^1^T_1u_, and 2^1^G_u_ to their ground vibrational levels at 3.12, 3.40, and 3.43 eV, respectively (Fig. 6). Hence, ISC from these singlet states at above 3.7 eV is expected to be outcompeted by VR. Alternatively, PO (Scheme (3)) is possible from 1^1^G_u_, 1^1^T_1u_, and 2^1^G_u_ vibronic states, if they transition to 2^1^H_u_ by internal conversion (IC) before VR to below 3.7 eV. Because both IC and ISC are slower than VR by an order of magnitude or more, PO from 1^1^G_u_, 1^1^T_1u_, and 2^1^G_u_ vibronic states will be minor, but may not be negligible. In conclusion, the major PO is expected to be through direct excitation of 2^1^H_u_. Accordingly, *k*_pd_ spectrum (data points) in Fig. 5c follows the 2^1^H_u_ band. However, some deviation is seen, being highest for the 3.88 eV (320 nm) data point and over the 2^1^H_u_ Gaussian, suggestive of additional excitations contributing, possibly through 1^1^G_u_, 1^1^T_1u_, and 2^1^G_u_ as discussed above.

We illustrate Scheme (3) with the gray arrows in the Jablonski diagram of Fig. 6. First, C_60_ is photoexcited through 1^1^A_g_(^1^C_60_) → 2^1^H_u_(^1^${\mathrm{C}}_{60}^{{\ast}{\ast}}$). Then, ^1^${\mathrm{C}}_{60}^{{\ast}{\ast}}$ transitions to ^3^${\mathrm{C}}_{60}^{{\ast}{\ast}}$ (2^1^H_u_ → 2^3^H_u_) via ISC. Subsequently, triplet–triplet annihilation (TTA)^26^ occurs with sensitization of ^1^O_2_:$2^{3}{\mathrm{H}}_{\mathrm{u}}\bullet{\,}^{3}{\mathrm{O}}_{2}\mathop{\rightarrow}1^{1}{\mathrm{T}}_{\mathrm{1g}}\bullet{\,}^{1}{\mathrm{O}}_{2}$. TTA also leaves C_60_ at an excited singlet state (1^1^T_1g_), which can readily react with ^1^O_2_:$1^{1}{\mathrm{T}}_{\mathrm{1g}}\bullet{\,}^{1}{\mathrm{O}}_{2}\mathop{\rightarrow}{\mathrm{C}}_{60}{\mathrm{O}}$. Hence, both 1^1^T_1g_ and ^1^O_2_, two energetic species, are created at the same time and same place (in collision complex) and have a higher chance to react. Another useful interpretation is that a single photon’s energy ($h\upsilon$) is partially utilized in sensitizing ^1^O_2_ while the excess energy leaves C_60_ at an excited state, which can react with ^1^O_2_. Scheme (3) may be expressed in more detail as:

4 $$	{\,}^{1}{\mathrm{C}}_{60} + {\,}^{3}{\mathrm{O}}_{2} \to {\,}^{1}{\mathrm{C}}_{60} \bullet {\,}^{3}{\mathrm{O}}_{2}\mathop{\longrightarrow}\limits^{{\mathrm{UV}}}{\,}^{1}{\mathrm{C}}_{60}^{ \ast \ast } \bullet {\,}^{3}{\mathrm{O}}_{2}\mathop{\longrightarrow}\limits^{{\mathrm{ISC}}}\\ 	\hskip 25pt {\,}^{3}{\mathrm{C}}_{60}^{ \ast \ast }\bullet {\,}^{3}{\mathrm{O}}_{2}\mathop{\longrightarrow}\limits^{{\mathrm{TTA}}}{\,}^{1}{\mathrm{C}}_{60}^{\ast} \bullet {\,}^{1}{\mathrm{O}}_{2} \to {\mathrm{C}}_{60}{\mathrm{O}}$$

**Fig. 7:**
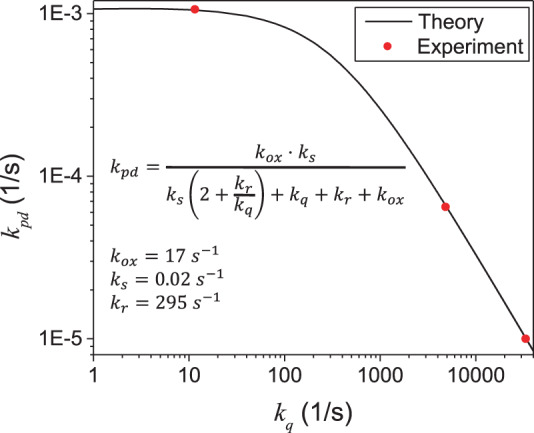
Fitting of theoretical *k*_pd_ expression to experimental data. The fitted values of *k*_ox_ and *k*_r_ are given in the inset.

We provide a mathematical analysis of PO kinetics for Scheme (3), which considers all the steps as well as reverse/competing processes, such as ^1^O_2_ quenching, relaxation of ${\mathrm{C}}_{60}^{\ast}$, and complex dissociations (see Supplementary Information; Kinetics Model). Despite the full complexity of this model, it predicts simply an exponential decay for [C_60_], being consistent with the measured kinetics. The model allows us to write *k*_pd_ as a function of ^1^O_2_ quenching rate, *k*_*q*_ = 1/*τ*. Fitting of this function to experimental data (Fig. 7) reveals the rate constants, *k*_ox_ and *k*_r_, associated with ${\mathrm{C}}_{60}^{\ast} \bullet {\,}^{1}{\mathrm{O}}_{2}\mathop { \to }\limits^{k_{\mathrm{ox}}} {\mathrm{C}}_{60}{\mathrm{O}}$ and ${\mathrm{C}}_{60}^{\ast} \bullet {\,}^{1}{\mathrm{O}}_{2}\mathop{\longrightarrow}\limits^{{k_r}}{\mathrm{C}}_{60} \bullet {\,}^{1}{\mathrm{O}}_{2}$, respectively, which has an interesting implication, as discussed below.

### Mechanism of C_60_ photooxidation in the atmosphere

PO of C_60_ is expected to be significantly accelerated in the atmosphere thanks to dramatically prolonged *τ* in the air (i.e., tens of minutes). Indeed, PO can dominantly occur as a two-photon process (Scheme (2), illustrated in Supplementary Fig. 5) driven by visible and UVA photons, being abundant in solar radiation. Unlike in solvents, it is challenging to monitor PO of C_60_ in air, since C_60_ being at detectable concentrations in air, would quickly undergo aggregation as well as adsorption to enclosure walls. However, *k*_pd_ can be predicted from the rate constants, *k*_ox_ and *k*_r_, which are already captured in the present work from C_60_ dispersions in solvents. As such, we compute *k*_pd_ = 0.011 s^−1^ for AM 1.5 solar radiation, suggesting a half-life of 63 s (see Supplementary Information; Kinetics Model). This rapid PO of C_60_ in the atmosphere potentially explains its scarceness in the environment^27^.

### Two excited singlets by triplet–triplet annihilation

By its description in the literature, TTA involves Dexter energy transfer from a triplet to another, after which the acceptor transitions to a higher energy state (singlet), while the donor returns to its ground singlet state. On the other hand, Scheme (3) engages a unique TTA, which produces two excited singlets simultaneously and thereby enables an efficient photochemistry. This scheme is stimulating for the conception of novel efficient photochemical processes, implemented with C_60_ or other photosensitizers.

## Methods

### C_60_ solution preparation

A stock solution of C_60_ was first prepared by dissolving 1 mg of C_60_ (Thermo Fisher Scientific, >99.9%) in 10 mL of solvent via ultra-sonication for 30 min. Next, the solution was kept undisturbed in the dark for 30 min to let insoluble aggregates (e.g., C_60_O) settle down. Subsequently, the supernatant was transferred by a pipette to a spectrophotometer cell (optical path length of 10 mm) filled with the same solvent until the absorbance of C_60_ at 256 nm reaches 1.42 (as monitored by a spectrophotometer), corresponding to C_60_ concentration of 5.67 μM. Finally, the prepared solution was stored in a sealed glass vial and kept in the dark before use. High purity of C_60_ in the prepared C_60_ solution is confirmed by mass spectrometry (Supplementary Fig. 2).

### Excitation spectroscopy for photooxidation

Photooxidation (photodecay) rate of C_60_, *k*_pd_, was measured as a function of excitation wavelength using the Fluorolog-3 spectrofluorometer. In a typical acquisition, 100 μL of C_60_ in CCl_4_ solution was placed in a standard microfluorescence cuvette (Science Outlet, 10 mm optical length, 0.35 mL capacity) and excited at selected wavelengths (from 250 to 420 nm, at 10 nm intervals) using a bandpass of 5 nm. The emission (^1^O_2_ phosphorescence) was parked at 1270 nm with a bandpass of 30 nm. At each excitation wavelength, the acquisition was performed 3 times and an unexposed sample was employed at each acquisition. The time-series phosphorescence intensity was collected in-situ at every 2 s with detector integration time of 2 s. Hence, the monochromatic optical beam of the spectrometer served as a dual probe simultaneously for measurement and exposure. For each excitation wavelength, *k*_pd_ was derived from the exponential decay rate of the phosphorescence intensity, quantifying the decay rate of C_60_ concentration (see Supplementary Fig. 3).

## Supplementary information

Supplementary Information

Peer Review File

## Data Availability

The data that support the findings of this study are available from the corresponding author upon request.
