# Supplementary material for: Single-photon oxidation of C60 by self-sensitized singlet oxygen
Source: Commun Chem. 2020 Jun 4;3:71. doi: 10.1038/s42004-020-0318-x (PMC9814575; doi:10.1038/s42004-020-0318-x)
Supplement: Supplementary file 1 — Supplementary Information [file 42004_2020_318_MOESM1_ESM.pdf]

Supplementary Information for

Single-photon oxidation of C_{60} by self-sensitized singlet oxygen

Linqi Zhang et al.

Supplementary Note 1

Photooxidation (PO) of C_{60} was first observed by Taylor *et al.*[1] They found undissolved reddish-brown deposit after exposing their freshly prepared C_{60} in benzene (open to air) to ultraviolet (UV) radiation (using a water-cooled medium-pressure silica-jacketed Hanovia insertion UV lamp). They also observed an extensive oxidation of C_{60} after 10-16 h of UV exposure. They credited this PO to oxidation by O_3 (e.g., ozonation of C_{60}). A year later, Creegan *et al.* photooxidized C_{60} in benzene and found C_{60} epoxide ($C_{60}O$) as the primary photoproduct.[2] Heymann *et al.* also claimed ozonation of C_{60}. They detected $C_{60}O$ as well as other C_{60}-adducts (i.e., $C_{60}O_2$, $C_{60}O_3$) when C_{60} was exposed to O_3 in toluene.[3] Seven years later, they identified fullerene ozonide (e.g., $C_{60}O_3$) as the reaction intermediate in C_{60} oxidation reaction. They reported C_{60} first reacted with an O_3 molecule and produced $C_{60}O_3$. Subsequently, $C_{60}O_3$ dissociated to a $C_{60}O$ and an O_2 molecule.[4] Ozonation of C_{60} in the ambient atmosphere (traceable amount of ozone) and ozone-enriched water was later reported by Murdianti *et al.*[5] and Fortner *et al.*,[6] respectively.

An alternative mechanism for C_{60} PO is the oxidation of C_{60} by $^1O_2$. Foote *et al.* found the $^1O_2$ sensitization quantum yield of C_{60} to be 0.96 ± 0.04 at 532 nm laser excitation, but 0.76 ± 0.05 at 355 nm excitation.[7] However, they could not provide an explanation for the lower sensitization yield at 355 nm. The same year, Wood *et al.*[8] also observed the degradation of C_{60} in benzene under UV radiation (using a 150 W mercury arc lamp) and found $C_{60}O_n$ (n=1 to 5) as photoproducts by mass spectrometry. Later, in 1993, Taliani *et al.*[9] reported detection of $C_{60}O_2$ after exposing a C_{60} film (5 μm thick) to an Ar^+ laser (λ=488 nm). To explain the formation of the photoproducts, they proposed the mechanism of $^1O_2$ addition to C═C in the C_{60} cage. However, this explanation has not been corroborated in terms of the photoproducts reported in the literature on the photoproducts.[2,8,10–12]

In the same year, Juha *et al.*[13] reported fast decomposition of C_{60} when radiated in hexane with a XeCl-excimer laser (308 nm). A year later, they identified the photoproduct to be $C_{60}O$ by liquid

chromatography.[10] In addition, they observed no reaction between ground state C_{60} with externally generated $^1O_2$ stream. Accordingly, they concluded C_{60} needed to be electronically excited in order to react with $^1O_2$. Given C_{60} is an excellent $^1O_2$ photosensitizer, it is reasonable to anticipate $^1O_2$ was photosensitized by C_{60} in Juha's work by laser excitation. However, no direct evidence was provided to this end.

Finally, a more compelling elucidation was contributed by Schuster *et al.*[12] They excited C_{60} by a Hanovia 450 W medium pressure Hg arc lamp (200-400 nm) in a $^1O_2$ environment (produced by thermolysis of endoperoxides) and found a large yield of $C_{60}O$. In contrast, a significantly lower yield of $C_{60}O$ was observed in the absence of $^1O_2$. This finding confirms the role $^1O_2$ plays in C_{60} oxidation, but the mechanistic details of the reaction remain unknown. Schuster *et al.* have proposed the mechanism of $C_{60}O$ formation to be the reaction of $^1O_2$ with the triplet state of C_{60}. However, this explanation is not plausible because the proposed reaction is spin forbidden.

Supplementary Methods

UV exposures. Four mL of C_{60} solution (preparation described in the Methods section) was transferred to a quartz optical cell (Starna Cells, 10 mm optical length, 4 mL capacity). Subsequently, the cell was sealed with a plastic cap using Parafilm. Then, the cell was exposed to UV irradiation at 3.74 mW/cm^2. The irradiation (spectrum centered at 310 nm as seen in the Figure 1c) was generated by a pair of tube lamps (Ultra-Violet Products, XX-15 series, 15 W each) and the UV intensity was measured by a digital UVA/UVB meter (General Tools UV513AB).

UV-Vis absorption spectroscopy. The absorption spectra of C_{60} were acquired with a Varian Cary 300 double-beam UV-Vis spectrophotometer. The spectra were measured before and during UV exposures (i.e., after each exposure interval) during photooxidation. Pure solvent was used as the reference. The spectra were recorded in the wavelength range from 200 to 800 nm with data intervals of 1 nm. The scan rate was set to 600 nm/min.

Phosphorescence spectroscopy. The $^1O_2$ phosphorescence spectra in solvents (i.e., C_6H_{14}, $CHCl_3$ and CCl_4) were acquired by Fluorolog-3 spectrofluorometer (Horiba Jobin Yvon) equipped with a LN2-cooled solid-state IR detector (DDS-Series) and a xenon lamp (450 W) as the excitation source. In a typical acquisition, 200 μl of C_{60} solution was placed in a standard micro fluorescence cuvette (Science Outlet, 10 mm optical length, 0.7 mL capacity). Pure solution spectrum was used as the baseline. The Fluorolog system automatically normalizes the signal intensity by the source (i.e., the xenon lamp) intensity, which is measured by the reference detector. The excitation wavelength was parked at 375 nm with bandpass of 5 nm. The incident power was measured as 1.9 mW with a power meter (Thorlabs, PM16-121). The emission was scanned from 1225 to 1325 nm using bandpass of 20 nm. The data interval was set to 1 nm. The signal was integrated for 15 s per data point for C_{60} in C_6H_{14} and $CHCl_3$ and 3s per data point for C_{60} in CCl_4.

Fourier transform infrared spectroscopy. A Bruker Alpha FTIR spectrometer, operating in the attenuated total reflection (ATR) mode was employed. In a typical acquisition, 10 μL of C_{60} solution (in $CHCl_3$) was spotted on the ATR diamond detector. However, for higher signal-to-noise, the spectrum of Figure 3b was measured after casting 50 μL of C_{60} solution on the detector. Spectra were acquired after complete evaporation of the solvent at a resolution of 4 cm^{-1} and 24 scans-to-average in the wavenumber range of 500-3500 cm^{-1}.

Excitation spectroscopy for phosphorescence (photosensitization) of $^1O_2$. The excitation spectrum of C_{60} for $^1O_2$ phosphorescence ($^1O_2$ photosensitization) was acquired by Fluorolog-3 spectrofluorometer. C_{60} dissolved in CCl_4 and pure CCl_4 were used as the sample (signal) and baseline, respectively. The sample and baseline liquids were enclosed in a quartz cuvette (Science Outlet, 10 mm optical length, 0.35 mL capacity) during the acquisition. The emission ($^1O_2$ phosphorescence) was parked at 1270 nm with bandpass of 30 nm. The excitation was scanned from 700 to 200 nm (i.e., to minimize photodegradation history) with bandpass of 5 nm. Data interval and detector integration time were set to 2 nm and 0.5 s, respectively. A longer integration time would have yielded a better signal-to-noise, but we comprised it to keep photodegradation limited. To estimate the amount of photodegradation in a scan, we acquired subsequent scans, indicating 7.4% photodegradation per scan.

Mass spectrometry. The purity of C_{60} in solution before UV exposure (as-received C_{60}) is checked by an LTQ-OrbitrapXL mass spectrometer (Thermo Fisher Scientific). Specifically, 250 μL of C_{60} solution (in $CHCl_3$) was infused into an electrospray chamber by a syringe pump at a flow rate of 30 μL/min. The MS spectrum was collected at the negative ion mode with negative ion spray voltage of 3500 V and ion transfer tube temperature of 300 °C. Nitrogen drying gas at 80 °C was supplied to evaporate the solvent. The scan range was set from 700 to 820 m/z and the maximum injection time was set to 100 ms.

$^1O_2$ phosphorescence at excitation wavelengths of 395 and 455 nm

Sensitization of $^1O_2$ at 395 and 455 nm excitations was confirmed by observing the $^1O_2$ phosphorescence at 1273 nm as shown in Supplementary Figure 1.

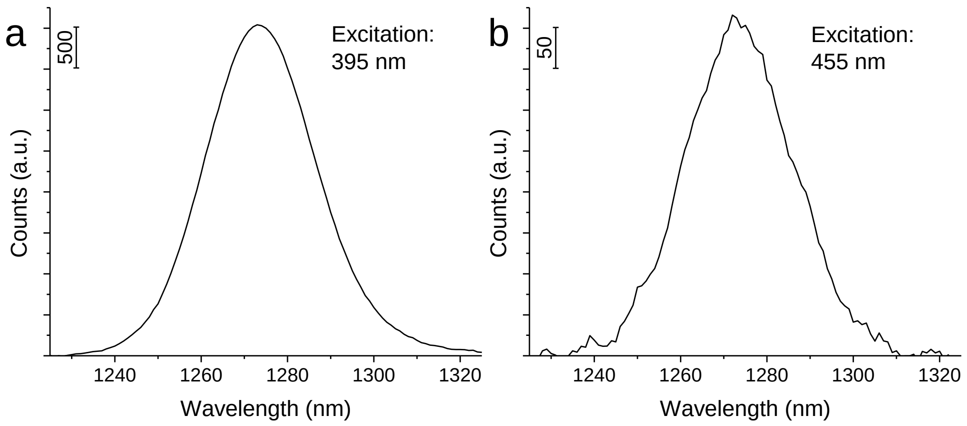

Supplementary Figure 1. Phosphorescence spectra of C_{60} (in CCl_4) under 395 (a) and 455 (b) nm radiation. The data interval and detector integration time are 1 nm and 4 s, respectively.

Mass spectrum of the as-received C_{60}

The mass spectrum of the as-received C_{60} is shown in Supplementary Figure 2. Only the base peak at 720 m/z was observed, confirming the high purity of C_{60} used in this work.

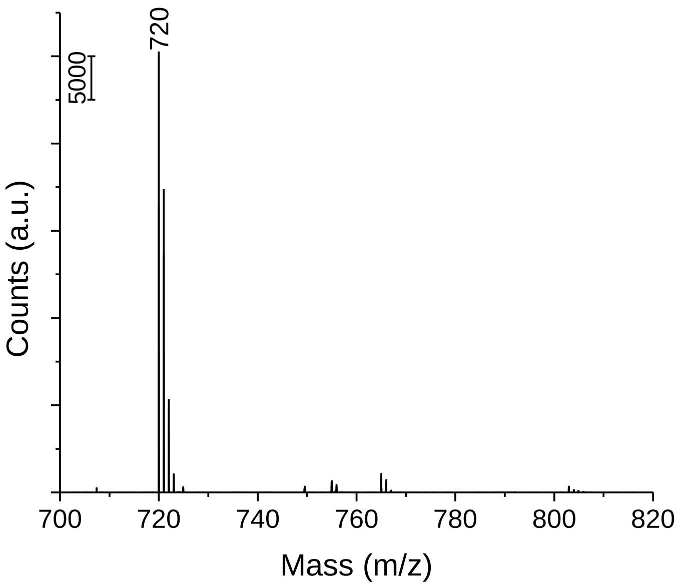

Supplementary Figure 2. Mass spectrum of the as-received C_{60}.

Kinetics of $^1O_2$ phosphorescence intensity

Two representative plots of $^1O_2$ phosphorescence intensity kinetics are shown in Supplementary Figure 3. These kinetics data were acquired under continuous excitation and signify decay of C_{60} by photooxidation. Supplementary Figure 3a is representative of the kinetics we observe for excitation wavelengths between 280 to 320 nm, where the intensity of $^1O_2$ emission shows an exponential-like decay indicating loss of C_{60} to oxidation products (e.g., $C_{60}O$). On the other hand, Supplementary Figure 3b is representative of the kinetics we observe for excitation wavelengths between 330 to 390 nm, where the emission intensity first shows an increase and then an exponential decay. Here, the monochromatic UV beam of the spectrophotometer served as excitation for both phosphorescence and photooxidation.

The normalized k_{pd} values (by photon counts (flux)) in Figure 5b was derived by the following steps: 1) fitting the natural log of the $^1O_2$ phosphorescence peak intensity values (red in Supplementary Figure 3) into a linear function (blue in Supplementary Figure 3); 2) dividing the slope of the fitted linear function by the beam photon counts (flux) at the corresponding excitation wavelength (measured by the reference detector equipped in the Fluorolog; 3) normalization of the k_{pd} values by the maximum k_{pd} (occurs at 300 nm excitation).

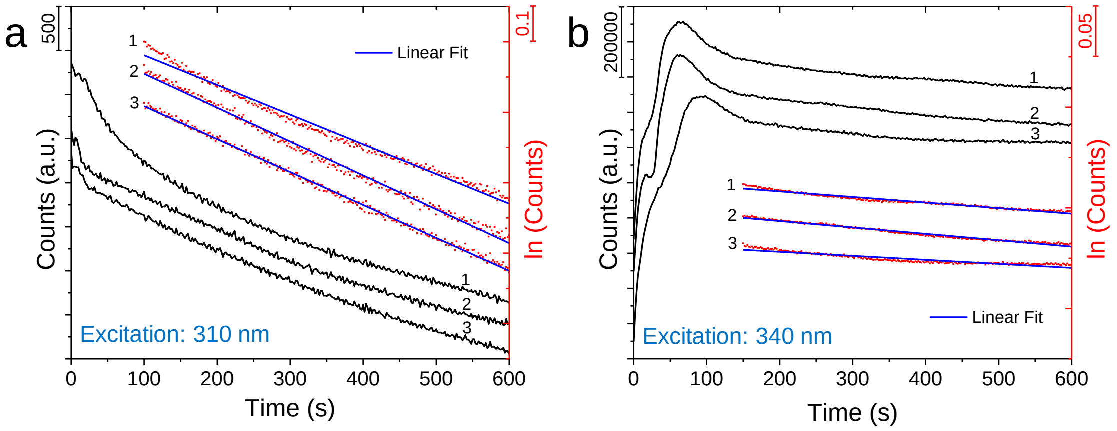

Supplementary Figure 3. Representative plots of $^1O_2$ phosphorescence intensity kinetics under continuous excitation of 310 nm (a) and 340 nm (b) wavelength. All 3 replications are shown. The plots are intentionally offset (separated) for clarity.

IR mode assignments for C_{60} photoproducts after 6 h of photooxidation

The detailed IR modes for C_{60} photoproducts are listed in Supplementary Table 1.

Supplementary Table 1. IR mode assignments of C_{60} photoproducts after 6 h of photooxidation

Peak Position [cm^{-1}]	Assignments	Peak Position [cm^{-1}]	Assignments
701, 791	CH_2 rocking	1462	C─H scissoring
864	C─H out of plane bending	1634, 1659	C═O stretching (cyclic alkene)
1013, 1084	C─O stretching (alcohol)	1730	C═O stretching (aldehyde)
1258	C─O stretching (ether)	2853	C─H symmetric stretching (alkane)
1378	C─H rocking (methyl)	2922, 2960	C─H asymmetric stretching (alkane)
1412	C─H bending (aliphatic chain)	3210, 3367	O─H stretching (alcohol)

Kinetics Model

Calculation of O_2 to C_{60} ratio

The mole fraction solubility of a gas in a solvent, x_g, is defined as[14]

$$x_g = \frac{n_g}{n_g + n_s} \tag{1}$$

where n_g is the maximum number of moles of the gas soluble in n_s moles of the solvent.

x_g of O_2 in C_6H_{14}, $CHCl_3$ and CCl_4 are given as 2.26×10^{-3}, 0.73×10^{-3} and 1×10^{-3} in the literature.[14–16] Taking 1 L of the solvent, we compute n_s using the density and molar mass of the solvent from the literature. Then, n_g is computed from Supplementary Equation 1 which also equals the molar concentration of the gas. Accordingly, the O_2 concentration in C_6H_{14}, $CHCl_3$ and CCl_4 are calculated as 1.72×10^{-2}, 9.12×10^{-3} and 1.03×10^{-2} M, respectively. The default C_{60} concentration is 5.67 μM in all solvents (Methods). Hence, the O_2 to C_{60} ratio in C_6H_{14}, $CHCl_3$ and CCl_4 are computed as 3034:1, 1608:1 and 1817:1, respectively.

Estimation of the transition (optical pumping) rate for $1^1A_g \rightarrow 2^1H_u$

The pumping rate, k_p, can be derived from:

$$P(\upsilon) = \sigma_{abs}(\upsilon) \cdot I(\upsilon) = k_p(\upsilon) \cdot h\upsilon \tag{2}$$

where P is the power absorbed by a single C_{60} molecule, σ_{abs} is its absorption cross section, I is the radiation intensity, and $h\upsilon$ is the photon energy. We will also use the relationship between the absorption cross section and molar attenuation coefficient, ε, as:

$$\sigma_{abs} = 3.8\times10^{-21}\varepsilon\ (\sigma_{abs}\ in\ cm^2\ and\ \varepsilon\ in\ M^{-1}cm^{-1}) \tag{3}$$

Hence,

$$k_p(\upsilon) = \frac{3.8\times10^{-21}\varepsilon(\upsilon)\cdot I(\upsilon)}{h\upsilon} \tag{4}$$

For the exact computation of k_p, Supplementary Equation 4 should be integrated over the energy range of the 1 $^1A_g \longrightarrow 2\ ^1H_u$ transition, I being per photon energy. However, resolving ε for 1 $^1A_g \longrightarrow 2\ ^1H_u$ is difficult as it overlaps with multiple and higher oscillator strength transitions. Here, we need a conservative estimate of k_p for validating various assumptions and approximations in the following section. As will be discussed in the section, a sufficiently low value of k_p is needed. Therefore, for a conservative approximation, we adopt an overestimated average value of ε and simplify the integration to a multiplication (Supplementary Equation 4), using the integrated value of I, $I = 3.74\ mW/cm^2$. Accordingly, we pick ε of C_{60} at $h\upsilon = 4.0\ eV$ ($\varepsilon = 1.2 \times 10^4\ M^{-1}cm^{-1}$).[17] This value of ε is conservative (overestimated), because photon energy of $h\upsilon = 4.0\ eV$ ($\lambda = 310\ nm$) is close to the center (maximum) of the 1 $^1A_g \longrightarrow 2\ ^1H_u$ absorption band (i.e., $\lambda = 299\ nm$). Furthermore, additional transitions contribute to ε at $h\upsilon = 4.0\ eV$. Accordingly, k_p is computed as $0.27\ s^{-1}$.

Single-photon oxidation of C_{60} with self-sensitized $^1O_2$ (Scheme 3)

Supplementary Figure 4 is a detailed mechanistic illustration of the single-photon oxidation of C_{60} with self-sensitized $^1O_2$ (Scheme 3) as unraveled in the present work. The arrows in the illustration depict the instrumental physical and chemical processes involved in Scheme 3 and are labeled with their rate constants. The initial, final and transient species involved in these processes are also shown. Among those species, 5 are included in our mathematical below. Their concentrations are denoted by c, w, x, y, z, as indicated in parentheses. Further details of Scheme 3 and its mathematical formulation are given below.

In the first step of Scheme 3, a collision complex, $C_{60}{\bullet}^3O_2$, forms. A ground state C_{60} collides with a ground state oxygen, $^3O_2$, forming a collision complex with reaction rate constant of K:

$$C_{60} + {}^3O_2 \xrightarrow{K} C_{60}{\bullet}\ {}^3O_2 \quad (5)$$

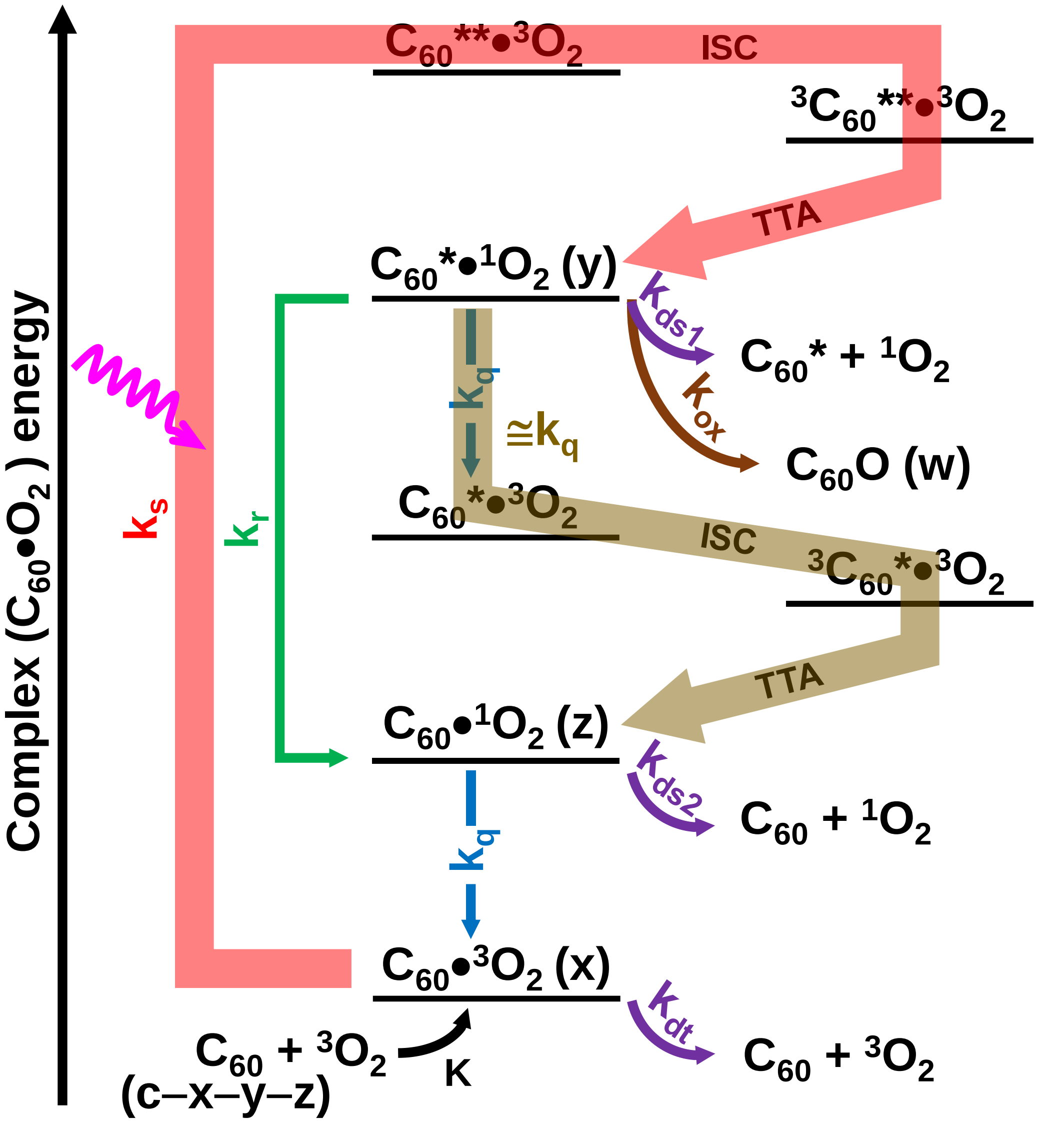

Supplementary Figure 4. Mechanistic illustration of Scheme 3.

Second, C_{60} of the collision complex is photoexcited, sensitizing $^1O_2$ and forming $^1C_{60}{*}{\bullet}{}^1O_2$, as described by Supplementary Equation 6. As discussed in the main text, this process actually involves the following subsequent steps: i) optical absorption, creating $^1C_{60}{**}{\bullet}{}^3O_2$; ii) intersystem crossing to $^3C_{60}{**}{\bullet}{}^3O_2$; iii) photosensitization of $^1O_2$ by triplet-triplet annihilation resulting in $^1C_{60}{*}{\bullet}{}^1O_2$. The asterisks denote excited states, ** indicating the higher energy excited state. The three steps are lumped to a single rate constant of k_s. ('s' for sensitization). In terms of radiation intensity, I, and absorption crossection, σ_{abs},

photon energy, $h\upsilon$, and photosensitization quantum yield, Φ_s, $k_s(\upsilon) = (I(\upsilon)\sigma_{abs}(\upsilon)/h\upsilon)\Phi_s(\upsilon)$. Note that uppercase K in Supplementary Equation 5 is the reaction rate constant, while in the remainder of this derivation we will introduce only rate constants, which are all denoted by lowercase k's.

$$C_{60}\bullet\ ^3O_2 + h\upsilon \xrightarrow{k_s} C_{60}{}^*\bullet\ ^1O_2 \tag{6}$$

Finally, C_{60} and O_2, both excited, react, as given by Supplementary Equation 7 below. The excited complex, $C_{60}{}^*\bullet{}^1O_2$, transforms to $C_{60}O$ with rate constant of k_{ox} ('ox' for oxidation).

$$C_{60}{}^*\bullet\ ^1O_2 \xrightarrow{k_{ox}} C_{60}O + \frac{1}{2}O_2 \tag{7}$$

The rate of $C_{60}O$ formation is governed by Supplementary Equation 8, where $\dot{w}$ is the rate of increase of $C_{60}O$ concentration; and y is the concentration of $C_{60}{}^*\bullet{}^1O_2$.

$$\dot{w} = k_{ox} \cdot y \tag{8}$$

$C_{60}{}^*\bullet{}^1O_2$ has four decay pathways: i) $C_{60}{}^*$ and $^1O_2$ react and yield $C_{60}O$ (Supplementary Equation 7); ii) $^1O_2$ in the complex is quenched by the surrounding solvent at a quenching rate constant of k_q. Hence, $C_{60}{}^*\bullet{}^1O_2$ converts to $C_{60}{}^*\bullet{}^3O_2$. Subsequently, $C_{60}{}^*\bullet{}^3O_2$ converts to $C_{60}\bullet{}^1O_2$ after intersystem crossing and triplet-triplet annihilation. The complete process may be described by: $C_{60}{}^*\bullet\ ^1O_2 \xrightarrow{k_q} C_{60}{}^*\bullet\ ^3O_2 \xrightarrow{ISC+TTA} C_{60}\bullet\ ^1O_2$. Here, the second step is faster by 3 orders of magnitude or more.[7] Therefore, the decay pathway may be described shortly by: $C_{60}{}^*\bullet\ ^1O_2 \xrightarrow{k_q} C_{60}\bullet\ ^1O_2$; iii) $C_{60}{}^*\bullet{}^1O_2$ converts to $C_{60}\bullet{}^1O_2$, where $C_{60}{}^*$ electronically relaxes to C_{60} with a transition rate of k_r: $C_{60}{}^*\bullet\ ^1O_2 \xrightarrow{k_r} C_{60}\bullet\ ^1O_2$; iv) $C_{60}{}^*\bullet{}^1O_2$ dissociates to $C_{60}{}^*$ and $^1O_2$ via $C_{60}{}^*\bullet\ ^1O_2 \xrightarrow{k_{ds1}} C_{60}{}^* +\ ^1O_2$ at a dissociation rate constant of k_{ds1}. Therefore, the time rate of change of $C_{60}{}^*\bullet{}^1O_2$ concentration, $\dot{y}$, is governed by:

$$\dot{y} = k_s x - k_q y - k_r y - k_{ox} y - k_{ds1} y \tag{9}$$

where x is the concentration of $C_{60}{\bullet}^3O_2$.

As discussed in the main text, $C_{60}{*}{\bullet}^1O_2$ formation (Supplementary Equation 6) involves the specific transition of $1\ ^1A_g \longrightarrow 2\ ^1H_u$ ($C_{60} \rightarrow C_{60}^{**}$ or $C_{60}{\bullet}\ ^3O_2 + h\upsilon \rightarrow C_{60}^{**}{\bullet}\ ^3O_2$), whose theoretical photon energy threshold is 3.72 eV (experimentally, we measure the photooxidation threshold at 3.7 eV, being remarkably close to 3.72 eV, and that is how we assign the associated transition to $1\ ^1A_g \longrightarrow 2\ ^1H_u$). For lower energy transitions (e.g., $1\ ^1A_g \longrightarrow 1\ ^1T_{1g}$), $C_{60}{\bullet}^1O_2$ forms. Accordingly, we associate those transitions with a different sensitization rate constant, k_{s2}, (i.e., $C_{60}{\bullet}\ ^3O_2 + h\upsilon \xrightarrow{k_{s2}} C_{60}{\bullet}\ ^1O_2;\ h\upsilon < 3.7\ eV$). It is possible that $C_{60}{\bullet}^1O_2$ is subsequently photoexcited with a second photon before $^1O_2$ is quenched and $C_{60}{*}{\bullet}^1O_2$ forms and subsequently oxidation occurs (Supplementary Equation 7). This oxidation pathway is represented by Scheme 2 of the main text. However, the probability of this event is negligibly low if the pumping rate, k_p ($h\upsilon \geq 3.7\ eV$) or k_{p2} ($h\upsilon < 3.7\ eV$) is sufficiently lower than the $^1O_2$ quenching rate, k_q. As computed conservatively (overestimated but not underestimated) by Supplementary Equation 4, $k_p = 0.27\ s^{-1} \ll k_q$ in all three solvents. Additionally, we have $k_{p2} \ll k_p$, because our excitation source is limited below the photon energy of 3.7 eV (Figure 1c). As a result, $k_{p2} \ll k_q$ and photooxidation due to excitation of $C_{60}{\bullet}^1O_2$ is insignificant. In conclusion, we encounter insignificant two-photon oxidation for photon energies below 3.7 eV.

Additionally, for the experimental conditions we employ in our investigation, two-photon oxidation of C_{60} for photon energies above 3.7 eV is ignorable, too. The excitation source employed in our study is characterized with a major narrow UV band peaking at 310 nm (Figure 1c) which ideally favors Scheme 3. Theoretically, however, two-photon oxidation is not impossible under this excitation. The intermediate, $C_{60}{\bullet}^1O_2$, can be excited to $C_{60}^{**}{\bullet}^1O_2$ (i.e., C_{60} is excited as $1\ ^1A_g \longrightarrow 2\ ^1H_u$), which is expected to undergo reaction and yield $C_{60}O$. However, for the solvents we employ, even the longest lifetime of $C_{60}{\bullet}^1O_2$ (0.087 s in CCl_4) is significantly shorter than the period between two subsequent

excitations of C_{60}, being 3.7 s ($1/k_q$). Therefore, we ignore two-photon oxidation for photon energies above 3.7 eV. In conclusion, we ignore all two-photon processes in explanation of our experimental kinetics data. The main text also provides experimental evidence, ruling out two-photon oxidation.

On the other hand, concentration of C_{60}•$^1O_2$, z, may not be ignorable. As discussed above, C_{60}•$^1O_2$ may be generated through the pathways: $C_{60}{}^{*}\bullet\ {}^1O_2 \overset{k_q}{\rightarrow} C_{60}\bullet\ {}^1O_2$ and $C_{60}{}^{*}\bullet\ {}^1O_2 \overset{k_r}{\rightarrow} C_{60}\bullet\ {}^1O_2$. Whereas, C_{60}•$^1O_2$ can be quenched as: $C_{60}\bullet\ {}^1O_2 \overset{k_q}{\rightarrow} C_{60}\bullet\ {}^3O_2$. The C_{60}•$^1O_2$ can also dissociate to C_{60} and $^1O_2$ via $C_{60}\bullet\ {}^1O_2 \overset{k_{ds2}}{\longrightarrow} C_{60} +\ {}^1O_2$ at a dissociation rate constant of k_{ds2}. Accordingly, time rate of change of z may be written as:

$$\dot{z} = (k_q + k_r)y - k_q z - k_{ds2} z \tag{10}$$

Note that we do not have optical excitation terms for C_{60}•$^1O_2$, because its optical excitation is low probability as discussed above (i.e., two-photon processes are ignored).

As for C_{60}•$^3O_2$, its population is determined by the following processes: i) it is generated by collision of C_{60} and $^3O_2$ (Supplementary Equation 5); ii) it is generated by solvent quenching of $^1O_2$ to $^3O_2$ from $C_{60}\bullet\ {}^1O_2 \overset{k_q}{\rightarrow} C_{60}\bullet\ {}^3O_2$; iii) C_{60}•$^3O_2$ is converted to the C_{60}*•$^1O_2$ by photosensitization; iv) it dissociates to C_{60} and $^3O_2$ via reaction: $C_{60}\bullet\ {}^3O_2 \overset{k_{dt}}{\longrightarrow} C_{60} +\ {}^3O_2$ with dissociation rate constant of k_{dt}. Therefore, the rate of change of C_{60}•$^3O_2$ concentration, $\dot{x}$, is governed by:

$$\dot{x} = K[C_{60}][\,{}^3O_2] + k_q z - k_s x - k_{dt} x$$

It may be rearranged to:

$$\dot{x} = K(c - x - y - z)[\,{}^3O_2] + k_q z - (k_s + k_{dt})x \tag{11}$$

where c is the concentration of unreacted C_{60} (in complex and free forms); that is, $c = [C_{60}] + x + y + z$. The total concentration of C_{60} in all forms, reacted as well as not reacted, remains constant during the initial stage of PO, where C_{60} is intact. Hence, $c + w = constant$. Accordingly,

$$\dot{w} = -\dot{c} \tag{12}$$

In this kinetics model for Scheme 3, we ignore the transient populations of $C_{60}{*}{\bullet}{}^3O_2$ (or $C_{60}{**}{\bullet}{}^3O_2$) and ${}^3C_{60}{*}{\bullet}{}^3O_2$ (or ${}^3C_{60}{**}{\bullet}{}^3O_2$). $C_{60}{*}{\bullet}{}^3O_2$ quickly (i.e., ~ ns; e.g., the lifetime of S_1 state of C_{60} is 1.3 ns)[18] transforms to ${}^3C_{60}{*}{\bullet}{}^3O_2$. Subsequently, ${}^3C_{60}{*}{\bullet}{}^3O_2$ (or ${}^3C_{60}{**}{\bullet}{}^3O_2$) rapidly (~ ns, for example, the lifetime of T_1 state of C_{60} is 330 ns in air saturated C_6H_6)[7] undergoes triplet-triplet annihilation and converts to $C_{60}{*}{\bullet}{}^1O_2$ (or $C_{60}{**}{\bullet}{}^1O_2$). Hence, accumulation of such populations is ignored.

Next, we will solve for Supplementary Equations 9, 10, and 11 under the local steady-state approximation. This approximation holds when rate of change of a population concentration is sufficiently lower than the number of reactions/transitions occurring per unit time per unit volume. In other words, the generation and annihilation rates approximately (but not exactly) balance each other and they are sufficiently larger than the rate of change of the population. The approximation is local in time. With this approximation, $\dot{y}$, $\dot{z}$, and $\dot{x}$ are set to 0 in Supplementary Equations 9, 10 and 11. Thus,

$$\dot{y} = k_s x - \left(k_q + k_r + k_{ox} + k_{ds1}\right) y = 0 \tag{13}$$

$$\dot{z} = (k_q + k_r) y - k_q z - k_{ds2} z = 0 \tag{14}$$

$$\dot{x} = K(c - x - y - z)\left[\,{}^3O_2\right] + k_q z - (k_s + k_{dt}) x = 0 \tag{15}$$

First, we solve for z and x in terms of y in Supplementary Equations 14 and 15 and then substitute them in Supplementary Equation 13 as follows:

$$z = \frac{k_q + k_r}{k_q + k_{ds2}} y$$

$$\left(k_s + k_{dt} + \mathrm{K}[\,^3O_2]\right)\mathrm{x} = Kc[\,^3O_2] + \left(\frac{k_q(k_q + k_r)}{k_q + k_{ds2}} - \mathrm{K}[\,^3O_2]\left(1 + \frac{k_q + k_r}{k_q + k_{ds2}}\right)\right)\mathrm{y}$$

$x = ac + by$, where $a = \frac{K[\,^3O_2]}{k_s + k_{dt} + \mathrm{K}[\,^3O_2]}$ and $\mathrm{b} = \frac{\left(\frac{k_q(k_q+k_r)}{k_q+k_{ds2}} - \mathrm{K}[\,^3O_2]\left(1+\frac{k_q+k_r}{k_q+k_{ds2}}\right)\right)}{k_s+k_{dt}+\mathrm{K}[\,^3O_2]}$

Substituting into Supplementary Equation 13, $k_s\mathrm{ac} + k_s\mathrm{by} - \left(k_q + k_r + k_{ox} + k_{ds1}\right)\mathrm{y} = 0$. Hence,

$$y = \left(\frac{k_s a}{k_q + k_r + k_{ox} + k_{ds1} - k_s b}\right)\mathrm{c}$$

Substituting y into Supplementary Equation 8 and combining with Supplementary Equation 12 yields:

$$\dot{c} = -k_{ox}\mathrm{y} = -\left(\frac{k_{ox}k_s a}{k_q + k_r + k_{ox} + k_{ds1} - k_s b}\right)\mathrm{c} = -k_{pd}\mathrm{c} \qquad (16)$$

where $k_{pd} = \frac{k_{ox}\cdot k_s\cdot K[\,^3O_2]}{(k_q+k_r+k_{ox}+k_{ds1})(k_s+k_{dt}+K[\,^3O_2]) + k_s\left(K[\,^3O_2]\left(1+\frac{k_q+k_r}{k_q+k_{ds2}}\right) - \frac{k_q(k_q+k_r)}{k_q+k_{ds2}}\right)}$ is the photodecay rate constant. It is the exponential decay constant for [C_{60}].

Simplification of k_{pd} under the assumption of [C_{60}] << x and [C_{60}] << y

Based on the literature, photosensitization of $^1O_2$ by C_{60} occurs at a quantum yield, Φ_s, of close to unity in the visible range of the electromagnetic spectrum.[7,18] Φ_s is defined as the number of $^1O_2$ sensitized divided by number of photons absorbed by C_{60}. Hence, Φ_s being close to unity, means every photon absorption by C_{60} results in sensitization of a $^1O_2$. Accordingly, this result suggests every C_{60} must be already conjugated with a single or multiple O_2 molecule(s), unless the calculation is based on only the C_{60}, which is conjugated with $^3O_2$ (i.e. $C_{60}\bullet{}^3O_2$). However, this detail is not provided in those references,[7,18] and the calculations quantify the concentration of C_{60} from its as-measured (i.e., total) absorption coefficient. Therefore, one may conservatively make the assumption: $[C_{60}] \ll x$ and $[C_{60}] \ll y$. This condition also implies: $k_{dt} \ll K[\,^3O_2]$, $k_{ds1} \ll K[\,^3O_2]$ and $k_{ds2} \ll K[\,^3O_2]$.

Under this assumption, the expression for k_{pd} above simplifies by approximation. First, from Supplementary Equations 14 and 15 we have

$$K\frac{[C_{60}]}{x}[\,^3O_2] = (k_s + k_{dt}) - \frac{k_q(k_q+k_r)y}{(k_q+k_{ds2})x} \tag{17}$$

Supplementary Equation 17 is valid for $y/x \ll 1$ as well as $y/x = 1$ (i.e., presaturation, early saturation, or saturation). Note that, saturation occurs when excessive optical pumping of C_{60} takes place to the degree that $y/x \gg 1$. Here, only k_s is directly dependent on the pumping rate. On the other hand, the rate constants k_r, k_{ds2} and k_{dt} are independent of the intensity of incident radiation to a first degree. Similarly, $K[\,^3O_2]$ is constant with the pumping rate to a first degree, because in the solvents we employed, $[\,^3O_2]/[free\ and\ conjugated\ C_{60}] > 1000$ (see Calculation of O_2 to C_{60} ratio). Hence, the solvent may be treated as a reservoir of $^3O_2$. Given the assumption $[C_{60}]/x \ll 1$, then

$$K[\,^3O_2] \gg (k_s + k_{dt}) - \frac{k_q(k_q+k_r)y}{(k_q+k_{ds2})x} \tag{18}$$

For $y/x \ll 1$ (i.e., presaturation), the Inequality (Supplementary Equation 18) suggests

$$K[\,^3O_2] \gg (k_s + k_{dt}) \tag{19}$$

For $y/x = 1$ (i.e., early saturation) the righthand side of Inequality (Supplementary Equation 17) must still be positive. Hence, $(k_s + k_{dt}) > \frac{k_q(k_q+k_r)}{(k_q+k_{ds2})}$. Hence, from Inequality (Supplementary Equation 19), we also infer

$$K[\,^3O_2] \gg \frac{k_q(k_q+k_r)}{(k_q+k_{ds2})} \text{ and } K[\,^3O_2]\left(1+\frac{k_q+k_r}{k_q}\right) \gg \frac{k_q(k_q+k_r)}{(k_q+k_{ds2})} \tag{20}$$

Using Supplementary Equation 19 and 20 besides ignoring k_{ds1} and k_{ds2}, k_{pd} may be approximated as:

$$k_{pd} \cong \frac{k_{ox}\cdot k_s\cdot K[\,^3O_2]}{(k_q+k_r+k_{ox})(\cong K[\,^3O_2]) + k_s(\cong K[\,^3O_2]\left(1+\frac{k_q+k_r}{k_q}\right))} \cong \frac{k_{ox}\cdot k_s}{k_s\left(2+\frac{k_r}{k_q}\right)+k_q+k_r+k_{ox}} \tag{21}$$

Indeed, the Approximation (Supplementary Equation 21) can also be derived from Supplementary Equation 13. Under the assumption that $[C_{60}] \ll x$, $[C_{60}] \ll y$ and $[C_{60}] \ll z, x + y + z \cong c$. Hence, ignoring k_{ds1} and k_{ds2} and substituting $x \cong c - y - z$ in Supplementary Equation 13,

$$k_s(\mathrm{c} - \mathrm{y} - \frac{k_q + k_r}{k_q}\mathrm{y}) - \left(k_q + k_r + k_{ox}\right)y \cong 0$$

$$y \cong \frac{k_s}{k_s\left(2+\frac{k_r}{k_q}\right)+k_q+k_r+k_{ox}}c \qquad (22)$$

Hence, substituting (Supplementary Equation 22) in Supplementary Equation 16, it is approximated as:

$$\dot{c} = -k_{ox}y \cong -\frac{k_{ox}k_s}{k_s\left(2 + \frac{k_r}{k_q}\right) + k_q + k_r + k_{ox}}c$$

from which it follows $k_{pd} \cong \frac{k_{ox} \cdot k_s}{k_s\left(2+\frac{k_r}{k_q}\right)+k_q+k_r+k_{ox}}$, being same as Supplementary Equation 21.

Apparently, Supplementary Equation 21 has a singularity at $k_q = 0$, for which k_{pd} tends to zero, because of the term $k_s\left(2 + \frac{k_r}{k_q}\right)$ in the denominator tends to infinity. This situation is controversial, because k_{pd} is expected to be maximized due to infinite lifetime of $^1O_2$. The discrepancy is due to the exclusion of two-photon processes, which is a valid assumption under the conditions employed in the current investigation. Under this assumption, the extinction term for $C_{60}{\bullet}^1O_2$ (i.e., $[C_{60}{\bullet}^1O_2] = z$) in Supplementary Equation 14 is only $k_q z$, once optical pumping of $C_{60}{\bullet}^1O_2$ (to $C_{60}{*}{\bullet}^1O_2$) with a second photon is neglected. Therefore, when k_q tends to zero, there is no extinction of $C_{60}{\bullet}^1O_2$. Hence, all $C_{60}{\bullet}^3O_2$ will convert to $C_{60}{\bullet}^1O_2$ and all $C_{60}{\bullet}O_2$ will accumulate in the form of $C_{60}{\bullet}^1O_2$ with infinite lifetime of $^1O_2$. As a result, photooxidation (Scheme 3) will stop. In reality, the two-photon oxidation is non-zero, and increases with decreasing k_q. Once it is included in the model, the term $k_s\left(2 + \frac{k_r}{k_q}\right)$ in the

denominator of Supplementary Equation 21 will be modified to $k_s\left(2+\frac{k_r}{k_q+k_p}\right)$, where k_p is the optical pumping rate constant for $C_{60}\bullet{}^1O_2$. Hence, the singularity at $k_q = 0$ will be removed. However, there will be significant other modifications in the expression for k_{pd}, if schemes 2 and 3 are considered in parallel.

Computation of k_r and k_{ox}

In this section, we will compute k_r and k_{ox} from the experimentally measured k_{pd} values. k_r and k_{ox} are the two unknowns in Supplementary Equation 21 and treated as constants. k_s can be computed from the intensity of the incident radiation, absorption coefficient of C_{60} and $^1O_2$ photosensitization quantum yield by C_{60}. We measured k_{pd} values for three solvents under the same incident excitation intensity. Therefore, k_s is also a constant. Finally, k_q is the inverse of the $^1O_2$ lifetime, τ, in the solvent and hence is the variable. Therefore, k_{pd} is a function of k_q. The two unknowns, k_r and k_{ox}, can be computed by solving two equations. Or, equivalently, they can be found as fitting parameters by fitting Supplementary Equation 21 to the experimentally measured curve of k_{pd} versus k_q. Here, we will pursue the second strategy.

First, k_s has to be obtained. To compute k_s, $k_s = (I\sigma_{abs}/h\upsilon)\Phi_s$, we will use the excitation spectrum in Figure 1c. Here, the normalized spectrum, $N(\lambda)$, is for radiation intensity per wavelength, λ. Hence, the actual intensity per wavelength is $A \cdot N(\lambda)$, where A is a constant. Our UV optometer integrates radiation from 280 to 400 nm, which is $I = 3.74\,mW/cm^2$ for our PO exposures. We use this measured intensity to find A, i.e., $A\int_{280\,nm}^{400\,nm} N(\lambda)d\lambda = I = 3.74\,mW/cm^2$.

Second, we convert $A \cdot N(\lambda)$ to function of photon energy in eV, $F(E)$. For this conversion, we have to consider the following energy balance in $d\lambda$ and dE. Also, $E = 1240/\lambda$ where E is in eV and λ is in nm. Accordinly, $dE = -(1240/\lambda^2)d\lambda$. and $|dE| = -(1240/\lambda^2)|d\lambda|$ Therefore,

$$A \cdot N(\lambda)|d\lambda| = F(E)|dE| = \frac{1240 \cdot F(E)}{\lambda^2}|d\lambda|$$

Hence,

$$F(E) = F\left(\frac{1240}{\lambda}\right) = \frac{A \cdot N(\lambda)}{1240}\lambda^2 \quad (23)$$

Then, k_s can be computed as:

$$k_s = \int_{3.70\ eV}^{4.43\ eV} F(E)\sigma_s(E)\frac{1}{E}dE \quad (24)$$

where $\sigma_s(E) = \sigma_{abs}(E)\Phi_s$ is the cross section for photosensitization. We actually deconvoluted $\sigma_s(E)$ as a Gaussian band in Figure 5b and also compared it with $k_{pd}(E)$ in Figure 5c. However, there the band was resolved from phosphorescence excitation spectrum. The band corresponds to $1\ {}^1A_g \longrightarrow 2\ {}^1H_u$ transition, responsible for oxidation of C_{60}. We normalize this band to $H(E) = e^{-a(E-4.15eV)^2}$ with $a = 21.34\ (eV)^{-2}$. To convert $H(E)$ to $\sigma_s(E)$, we will first convert it to molar photosensitization coefficient, $\varepsilon_s(E)$, being analogous to molar attenuation coefficient, $\varepsilon(E)$. To this end, in Figure 5a, we will compare the maximum phosphorescence counts for this band (i.e., at the peak = 299 nm), $P(299\ nm)$, with phosphorescence counts of the same curve at a different photon energy, for which ε_s is known. Conveniently, we will use the phosphorescence intensity at 406 nm, $P(406\ nm)$.

Then, $\varepsilon_s(299\ nm)/\varepsilon_s(406\ nm) = P(299\ nm)/P(406\ nm)$. However, at 406 nm, the quantum yield for photosensitization of ${}^1O_2$ by C_{60} is unity. Therefore, $\varepsilon_s(406\ nm)$, is equal to molar attenuation coefficient, $\varepsilon(406\ nm)$, which is $0.25\times10^4\ L \cdot mol^{-1} \cdot cm^{-1}$. Hence, from the well-known relation, $\sigma = 3.82 \times 10^{-21}\varepsilon$ (σ in cm^2 and ε in $L \cdot mol^{-1} \cdot cm^{-1}$), it follows:

$$\sigma_s(299\ nm) = 3.82 \times 10^{-21}\frac{P(299\ nm)}{P(406\ nm)}\varepsilon(406\ nm)$$

$$= 3.82 \times 10^{-21}(\frac{3645}{4875})(0.25 \times 10^4) = 7.14 \times 10^{-18}\ cm^2$$

Accordingly, $\sigma_s(E) = \sigma_s(299\ nm) \cdot H(E)$ and k_s now can be computed from Supplementary Equation 24 as:

$$k_s = \sigma_s(299\ nm) \int_{3.70\ eV}^{4.43\ eV} F(E)\, e^{-21.34(eV)^{-2}(E-4.15eV)^2} \frac{1}{E} dE$$

where $F(E)$ can be fitted to a Gaussian function: $F(E) = 6.25e^{-8.75(eV)^{-2}(E-3.97eV)^2}$.

Then, k_s is computed as:

$$k_s = 7.14 \times 10^{-18}\ cm^2 \times 6.25 \int_{3.70\ eV}^{4.43\ eV} e^{-8.75(eV)^{-2}(E-3.97eV)^2}\, e^{-21.34(eV)^{-2}(E-4.15eV)^2} \frac{1}{E} dE = 0.02\ s^{-1}$$

Finally, by fitting three data points, (k_q, k_{pd}), to Supplementary Equation 21, k_{ox} and k_r are found as: 17 and 295 s^{-1}, respectively. The fitting of $k_{pd}(k_q)$ to the experimental data is shown in Figure 7.

Oxidation of C_{60} with free $^1O_2$

Photooxidation of C_{60} via this scheme takes three major steps: i) photosensitization of $^1O_2$ by C_{60}; ii) accumulation of the produced $^1O_2$ in the solvent; and iii) oxidation of C_{60} after colliding with free $^1O_2$ in the solvent. In the first step, $^1O_2$ is sensitized by C_{60} as:

$$C_{60} + {}^3O_2 + h\upsilon \xrightarrow{K_s} C_{60} + {}^1O_2 \quad (25)$$

Here, the intermediate steps of $C_{60}\bullet{}^3O_2$ formation, photosensitization of $^1O_2$ (excitation of C_{60} to $^1C_{60}$*, intersystem crossing of $^1C_{60}$* to triplet state, $^3C_{60}$*, triplet-triplet annihilation of $^3C_{60}*\bullet{}^3O_2$ to $C_{60}\bullet{}^1O_2$) and dissociation of $C_{60}\bullet{}^1O_2$ to C_{60} and $^1O_2$ (i.e., release of $^1O_2$) are all lumped to the reaction rate constant of K_s. Hence, the generation rate of $^1O_2$ (concentration per unit time), G_s, is:

$$G_s = K_s[C_{60}][{}^3O_2][h\upsilon] \quad (26)$$

where $[h\upsilon]$ stands for optical pumping rate of C_{60} being proportional to the intensity of radiation. The produced $^1O_2$ accumulates in the solvent. The decay of $^1O_2$ is mainly due to solvent quenching and the quenching rate, k_q, is inverse of the $^1O_2$ lifetime, $k_q = 1/\tau$.

At steady state (in local time), the generation rate of $^1O_2$ is equal to its quenching rate. Therefore,

$$G_s = K_s[C_{60}][\,^3O_2][h\upsilon] = [\,^1O_2]/\tau$$

It may be rearranged to:

$$[\,^1O_2] = \tau K_s[C_{60}][\,^3O_2][h\upsilon] \tag{27}$$

The third step can occur in two ways. Either $^1O_2$ collides with an excited C_{60} and forms $^1C_{60}{*}\bullet{}^1O_2$, which thereafter reacts to $C_{60}O$, or it collides with ground state C_{60} forming $C_{60}\bullet{}^1O_2$, which is subsequently excited to $^1C_{60}{*}\bullet{}^1O_2$ and reacts. In both cases, the overall reaction is given by:

$$C_{60} + \,^1O_2 + h\upsilon \xrightarrow{K_{ox}} C_{60}O + \frac{1}{2}O_2 \tag{28}$$

The rate of change of C_{60} population (i.e., $\frac{d}{d_t}[C_{60}]$) follows as:

$$\frac{d}{d_t}[C_{60}] = -K_{ox}[C_{60}][\,^1O_2][h\upsilon] \tag{29}$$

Substituting Supplementary Equation 27 into Supplementary Equation 29 yields:

$$\frac{d}{d_t}[C_{60}] = -K_sK_{ox}\tau[\,^3O_2][C_{60}]^2[h\upsilon]^2 \tag{30}$$

Therefore, ‘oxidation with free $^1O_2$’ is excluded as a major pathway due to the fact that Supplementary Equation 30 is not consistent with the observed $[C_{60}]$ kinetics, which is exponential decay. Additionally, ‘oxidation with free $^1O_2$’ is expected to have quadratic dependence on radiation intensity (i.e., two

photons needed for the oxidation of a single C_{60} molecule), which is not supported by our experimental findings (i.e., Figure 4b).

The corresponding schemes for photooxidation by free $^1O_2$ is given by:

$$\text{i) } C_{60s}\bullet\,^3O_2 \overset{h\upsilon}{\rightarrow} C_{60s}\bullet\,^1O_2 \rightarrow C_{60s} + \,^1O_2; ii)\ C_{60} \overset{h\upsilon}{\rightarrow}\,^1C_{60}^* \overset{^1O_2}{\longrightarrow}\,^1C_{60}^*\bullet\,^1O_2 \rightarrow C_{60}O \tag{31}$$

$$\text{i) } C_{60s}\bullet\,^3O_2 \overset{h\upsilon}{\rightarrow} C_{60s}\bullet\,^1O_2 \rightarrow C_{60s} + \,^1O_2; ii)\ C_{60} + \,^1O_2 \rightarrow C_{60}\bullet\,^1O_2 \overset{h\upsilon}{\rightarrow}\,^1C_{60}^*\bullet\,^1O_2 \rightarrow C_{60}O \tag{32}$$

which we name as Scheme 4 and Scheme 5, respectively. Here, C_{60s} denotes the sensitizer (photocatalyst), unlike C_{60}, which is the reactant. The overall reaction can be written as:

$$C_{60s} + \,^3O_2 + C_{60} + 2h\upsilon \longrightarrow C_{60}O + \frac{1}{2}O_2 + C_{60s} \tag{33}$$

Hence, as also seen from Supplementary Equation 33, the forward reaction has quadratic dependence on both $[C_{60}]$ ($= [C_{60s}]$) and $[h\upsilon]$.

Additionally, 'oxidation with free $^1O_2$' is also ruled out as a major pathway by the following fundamental considerations. Schemes 4 and 5 are unfavorable in solvents for several reasons. First, $^1O_2$ is polar and C_{60} is highly polarizable. Hence, release of $^1O_2$ from C_{60} is hampered in nonpolar solvents after photosensitization due to dipole – induced dipole interaction (which on the other hand benefits the 'oxidation by self-sensitized $^1O_2$' pathway). Second, τ is too short. As a result, $^1O_2$ population accumulating in the solvent is limited. However, the major downfall of these schemes is that excited C_{60} and excited O_2 are not generated simultaneously by the same process as in our 'oxidation by self-sensitized $^1O_2$' scheme (Scheme 5). In Scheme 4, the lifetime of C_{60}* is about 1 ns, while its pumping rate is 0.27 s^{-1} ($1/k_p$). Hence, concentration of C_{60}* is inferred to be negligible and its collision with a $^1O_2$ is very unlikely in such short lifetime and low $^1O_2$ concentration. As for Scheme 5, τ must be long enough to allow complex formation ($C_{60}\bullet^1O_2$) and subsequent excitation of C_{60} resulting in $C_{60}^*\bullet^1O_2$. The longest τ in our study (~0.1 s in CCl_4) is significantly shorter than the period between two subsequent

excitations of C_{60} (~3.7 s). Hence, an excited O_2 and an excited C_{60} will hardly coincide in time and space.

On the other hand, such condition may be allowed in a gaseous ambient by prolonged τ, which is on the order of minutes. Oxidation of C_{60} by externally generated $^1O_2$ in gas phase and under radiation, as reported by Supplementary Reference 12, may therefore involve Scheme 5. Interestingly, however, excitation source employed in this work is the medium-pressure mercury lamp (200-400 nm UV radiation), which can also drive 'oxidation by self-sensitized $^1O_2$'.

Two-photon oxidation with self-sensitized C_{60} (Scheme 2)

Supplementary Figure 5 is a detailed mechanistic illustration of the two-photon oxidation of C_{60} with self-sensitized $^1O_2$ (Scheme 2). The illustration uses the same conventions as in Supplementary Figure 4.

Making the assumptions of: i) local steady state; ii) all C_{60} is conjugated with O_2; iii) ignoring k_{ds1} and k_{ds2}, Scheme 2 may be formulated as follows:

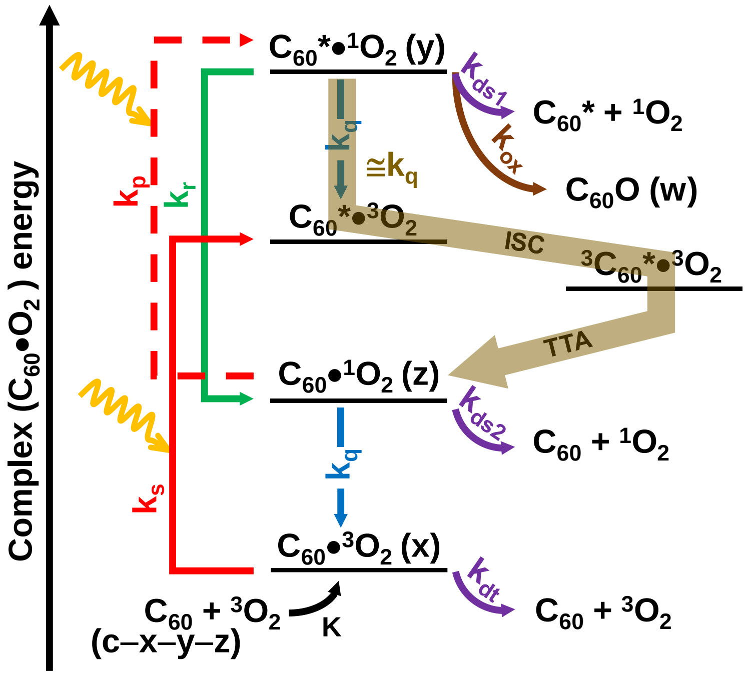

Supplementary Figure 5. Mechanistic illustration of Scheme 2.

$$\dot{y} = 0 = k_p z - (k_q + k_r + k_{ox})y \tag{34}$$

$$\dot{z} = 0 = k_s x + k_r y - (k_p + k_q)z \tag{35}$$

$$z = c - x - y \tag{36}$$

where k_p is the optical pumping rate constant for C_{60}.

From Supplementary Equation 34: $z = \frac{(k_q+k_r+k_{ox})}{k_p} y = ay$

From Supplementary Equation 35: $x = \frac{(k_p+k_q)a - k_r}{k_s} y = by$

Substituting in Supplementary Equation 36, $y = \frac{c}{1+a+b} = \frac{k_p k_s}{k_p k_s + (k_s+k_p+k_q)(k_q+k_r+k_{ox}) - k_p k_r} c$

$$\dot{c} = -k_{ox} y = -\frac{k_{ox} k_p k_s}{k_p k_s + (k_s + k_p + k_q)(k_q + k_r + k_{ox}) - k_p k_r} c$$

In addition, remembering $k_s = k_p \Phi_s$, it follows:

$$k_{pd} = \frac{k_{ox} {k_p}^2 \Phi_s}{{k_p}^2 \Phi_s + (k_p[1+\Phi_s] + k_q)(k_q+k_r+k_{ox}) - k_p k_r} \tag{37}$$

In our work, $k_s = 0.02\ s^{-1} \leq k_p \ll k_q$ for all three solvents studied. In this case, $k_{pd} \cong \frac{k_{ox} {k_p}^2 \Phi_s}{k_q(k_q+k_r+k_{ox})}$, having quadratic dependence on k_p. On the other hand, in air, k_q is ignorable with respect to other rate constants. Additionally, in atmosphere, visible and UVA photons have more abundance, for which $\Phi_s \cong 1$ (i.e., $k_s \cong k_p$). Obtaining the values of k_{ox} and k_r from the fitting of Supplemental Equation 21 (Figure 7), for atmosphere:

$$k_{pd} \cong \frac{k_{ox} k_s}{k_s + k_r + 2k_{ox}} = \frac{17 k_s}{k_s + 329} \tag{38}$$

where k values are in 1/s.

Estimation of k_{pd} in the atmosphere under solar radiation (based on Scheme 2)

Unlike in solvents, Scheme 2 may become significant in the atmosphere due to longer lifetime of $^1O_2$. In this section, we validate this expectation. As discussed in the above sections, Scheme 2 can be driven by both UV and visible photons. Solar irradiation impinging on Earth is 7% UV, 47% visible, and 46% IR.[19] For simplicity, we estimate k_{pd} based on the visible irradiation only, from 400 to 700 nm. Similar to Supplementary Equation 24, k_s can be computed as:

$$k_s = \int_{1.77\ eV}^{3.10\ eV} S(E)\sigma_{abs}(E)\frac{1}{E}dE \tag{39}$$

where $S(E)$ is the solar irradiation as a function of photon energy in eV and $\sigma_{abs}(E)$ is the absorption cross section of C_{60}. As in Supplementary Equation 23, the $S(E)$ can be converted from $T(\lambda)$ as follows:

$$S(E) = S\left(\frac{1240}{\lambda}\right) = \frac{T(\lambda)}{1240}\lambda^2 \tag{40}$$

where $T(\lambda)$ is the solar intensity per wavelength, which has been provided in the literature.[20] The $\sigma_{abs}(E)$ can be computed from the well-known relation, $\sigma_{abs}(E) = 3.82 \times 10^{-21}\varepsilon(E)$, where $\varepsilon(E)$ is the molar attenuation coefficient of C_{60} as a function of photon energy in eV. We obtain $\varepsilon(E)$ from Supplementary Reference 17.

To compute Supplementary Equation 39 numerically, we fit the experimental data for $S(E)$ and $\varepsilon(E)$ into two Gaussian functions: $S(E) = 587e^{-0.58(eV)^{-2}(E-1.34eV)^2}$ and $\varepsilon(E) = 1022e^{-6.77(eV)^{-2}(E-2.42eV)^2}$. The fits are shown in Supplementary Figure 6. Then, k_s can be computed as:

$$k_s = 3.82 \times 10^{-21}\int_{1.77eV}^{3.10\ eV} 587e^{-0.58(eV)^{-2}(E-1.34eV)^2} \times 1022e^{-6.77(eV)^{-2}(E-2.42eV)^2}\frac{1}{E}dE = 0.21\ s^{-1}$$

Accordingly, k_{pd} can be computed from Supplementary Equation 38 as: $k_{pd} \cong \frac{17k_s}{k_s+329} = 0.011\ s^{-1}$

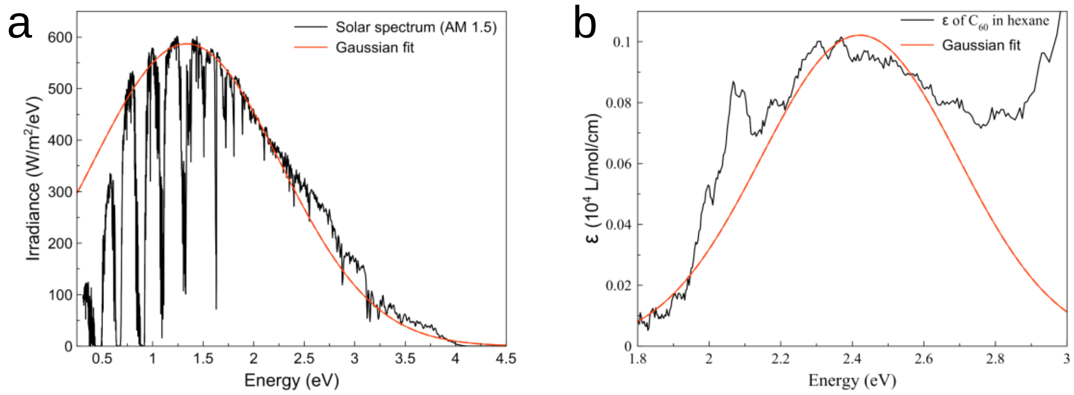

Supplementary Figure 6. a) AM 1.5 solar irradiation as a function of photon energy. b) Molar attenuation coefficient of C_{60} in hexane. The Gaussian fit, used in the numerical integration of k_s, is shown in red.

Supplementary Discussion

On the CCl_4-caused noise in the absorbance spectrum of C_{60} below 255 nm

As shown in Supplementary Figure 7, the absorbance of pure CCl_4 (solvent) overwhelms absorbance of C_{60} below 260 nm. As a result, the C_{60} peak gets obscured by the solvent, especially at wavelengths below 255 nm due to the attenuation of the optical beam. To check the accuracy of our absorbance at 260 nm for the C_{60} peak, we have acquired the absorption spectrum of C_{60} at a higher concentration (i.e., 14.76 μL), so that the contribution of C_{60} is enhanced in the raw data. As seen in Supplementary Figure 7, the C_{60} absorption peaks occur at the same wavelength for the two different concentrations. Hence, we validate the accuracy of our C_{60} absorbance values at 260 nm measured during degradation in CCl_4.

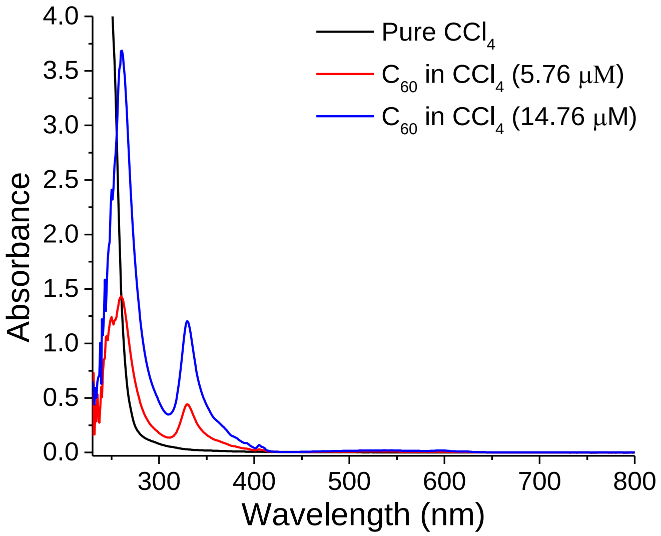

Supplementary Figure 7. Absorption spectra of CCl_4 and C_{60} in CCl_4 at different concentrations.

On the accuracy of the k_{pd} values derived from UV-Vis absorption spectra of C_{60}

The oxidation kinetics (Figure 4a), monitored from optical absorbance at 256 nm, suggest exponential decay of the C_{60} population through photooxidation. However, the time-series absorbance in Figure 4a, $A(\mathrm{t})$, is acquired from the remaining unreacted C_{60} molecules as well as the photoproduct (i.e., oxidized/photodegraded C_{60}). Therefore,

$$A(\mathrm{t}) = A(0)e^{-k_{pd}t} + A(\infty)\left(1 - e^{-k_{pd}t}\right) \tag{41}$$

where $A(0)$ and $A(\infty)$ are the absorbance of the sample before any oxidation (all C_{60}) and absorbance of the photoproduct after all C_{60} reacted (all photoproduct). Here, we assume the photoproduct's chemical composition is not changing with time. This assumption is reasonable at the earlier stage of the photodegradation. Supplementary Equation 41 may be rearranged to:

$$A(\mathrm{t}) = [A(0) - A(\infty)]e^{-k_{pd}t} + A(\infty) \tag{42}$$

Hence, $A(\mathrm{t})$ is an exponentially decaying function plus a constant. On the other hand, $\frac{d}{dt}A(\mathrm{t})$ is a purely an exponentially decaying function with the decay constant of k_{pd}:

$$\frac{d}{dt}A(\mathrm{t}) = -k_{pd}[A(0) - A(\infty)]e^{-k_{pd}t} \tag{43}$$

Therefore, k_{pd} can be derived more accurately from $\frac{d}{dt}A(\mathrm{t})$. k_{pd} is the slope of the line versus t. As an example, Supplementary Figure 8 shows extraction of k_{pd} for C_{60} in $CHCl_3$ from $\frac{d}{dt}A(\mathrm{t})$, where k_{pd} is found to be 6.77×10^{-5} being close to the k_{pd} value derived directly from $A(\mathrm{t})$ in Figure 4a (i.e., 6.47×10^{-5}). Hence, the photoproduct baseline could be ignored with negligible error and k_{pd} values derived from Figure 4a are reliable.

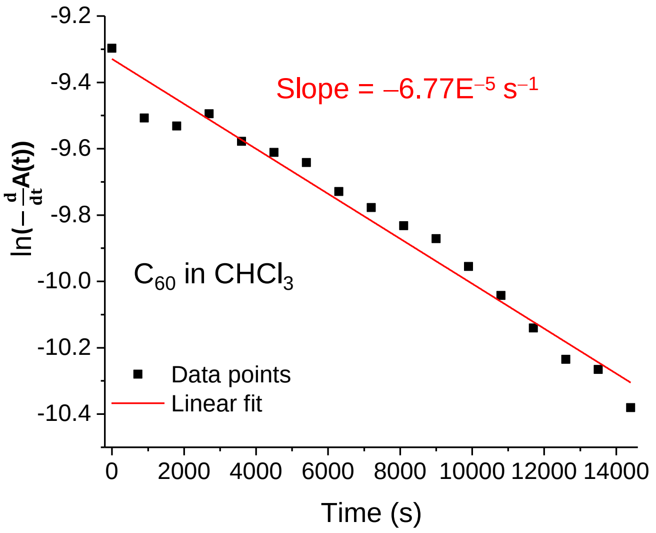

Supplementary Figure 8. Extraction of k_{pd} from $\frac{d}{dt}A(\mathrm{t},\lambda)$ for C_{60} in $CHCl_3$.

Validation of $^1O_2$ lifetimes adopted from the literature (for the $C_{60}\bullet{}^1O_2$ complex)

The $^1O_2$ lifetimes (τ) used in this work are taken from the literature, where $^1O_2$ was sensitized by other photosensitizers (i.e., tetraphenylporphine, rubicene, methylene blue)[21] instead of C_{60}. In this section, using the $^1O_2$ phosphorescence peak intensities from Figure 2a, we show those τ values are consistent and valid for $C_{60}\bullet{}^1O_2$.

The phosphorescence spectra of Figure 2a were acquired under excitation photon energy of $h\upsilon = 3.31\ eV$. From the Mathematical Model of Oxidation kinetics section, we have $C_{60}\bullet\ {}^3O_2 + h\upsilon \xrightarrow{k_{s2}} C_{60}\bullet\ {}^1O_2$ for $h\upsilon < 3.7\ eV$. $^1O_2$ in the $C_{60}\bullet{}^1O_2$ complex is quenched by the surrounding solvent at rate $k_q = 1/\tau$. Therefore, the time rate of change of $C_{60}\bullet{}^1O_2$ concentration, $\dot{z}$, is governed by:

$$\dot{z} = k_{s2}x - k_q z \tag{44}$$

The complex concentration, consisting of $C_{60}\bullet{}^3O_2$ (x) and $C_{60}\bullet{}^1O_2$ (z) is constant:

$$x + z = c \tag{45}$$

Substituting Supplementary Equation 45 into 44:

$$\dot{z} = k_{s2}c - k_{s2}z - k_q z \tag{46}$$

Using the local steady-state approximation, $\dot{z} = 0$:

$$\mathrm{z} = \mathrm{c}\frac{k_{s2}}{k_{s2}+k_q} \tag{47}$$

The $^1O_2$ phosphorescence intensity is proportional to z (i.e., number of $^1O_2$ to $^3O_2$ transitions (intersystem crossing) per unit time per unit volume equals $k_{ISC}z$, where k_{ISC} is the rate constant for intersystem crossing). Hence, we write:

$$Phosphorescence\ peak\ intensity \propto z = c\frac{k_{s2}}{k_{s2}+k_q}$$

$$Phosphorescence\ peak\ intensity = I_p(k_q) = A\frac{k_{s2}}{k_{s2}+k_q} \quad (48)$$

where A is a scaling factor. If k_q values, borrowed from the literature, are valid, then they should satisfy the relation, $I_p(k_q)$, given by Supplementary Equation 48. k_{s2} is calculated to be 0.4 s^{-1} from Supplementary Equation 4 using $\varepsilon = 0.6{\times}10^4\ M^{-1}cm^{-1}$ at $h\upsilon = 3.31$ eV. We measured phosphorescence peak intensity for all three solvents under the same incident excitation intensity of 9.5 mW/cm^2. A good match is obtained between Supplementary Equation 48 and (k_q, I_p) data points, by fitting A to 1200000 (Figure 2b). Hence, k_q values are validated.

Excitation spectra for phosphorescence and photosensitization of $^1O_2$

It should be mentioned that excitation spectra for phosphorescence and photosensitization of $^1O_2$ are not equivalent. However, in the present work, we consider the line shapes are the same per a given optical excitation (transition), as in the discussion of Figure 5. In particular, we focus on $1\ ^1A_g \longrightarrow 2\ ^1H_u$. Furthermore, we also assume the excitation line shape for photooxidation (of C_{60}) is also the same as that for phosphorescence (of $^1O_2$) per a given electronic transition. Here, we show these assumptions are exactly correct.

In excitation spectroscopy, the optical emission counts collected from a single molecule equal $I(v)\sigma_e(v)\Delta t/hv$, where I, σ_e, Δt and hv are radiation intensity, absorption cross section for emission, signal integration time and photon energy, respectively. Using Beer-Lambert law, the counts collected from a bulk sample are $NV(I(v)\sigma_e(v)\Delta t/hv)$, where N and V are the concentration and volume of the sample. Finally, the counts are normalized by the number of incident photons per signal integration, $I(v)A\Delta t/hv$, yielding $NL\sigma_e(v)$. Here A and L are the optical beam cross sectional area and path length (in the sample). In this analysis, I is assumed to be uniform in the sample. Hence, N should be sufficiently low or L should be sufficiently short, so that the excitation beam is minimally attenuated in the sample. Otherwise, the analysis should be corrected for decay of the beam intensity in the sample. In conclusion, the excitation spectrum for emission is $\sigma_e(v)$ multiplied by a constant.

Here, the emission is $^1O_2$ phosphorescence and $^1O_2$ belongs to the C_{60}•$^1O_2$ complex. Additionally, $\sigma_e(v) = \sigma_s(v)\Phi_e(v)$, where σ_s is the cross section for photosensitization of $^1O_2$ by C_{60} and $\Phi_e(v)$ is the probability of $^1O_2$ to decay (intersystem crossing to $^3O_2$) by phosphorescence. Clearly, if Φ_e has no spectral dependence (i.e., $\Phi_e(v) = constant$), then $\sigma_e(v)$ and $\sigma_s(v)$ have the same line shape. But, $\Phi_e(v) = k_e/(k_e + k_q + k_{ox}(v))$, where k's are the rate constants for $^1O_2$ emission (phosphorescence), solvent quenching (non-radiative decay) and oxidation, respectively from left to right. While k_e and k_q have no spectral dependence, k_{ox} has photon energy dependence, as our work shows. It becomes nonzero above the photon energy threshold of 3.7 eV. Nevertheless, when the excitation spectrum is deconvoluted to peaks, each peak is associated with a different optical excitation transition of C_{60}. (i.e., $S_o \longrightarrow S_n,\ n = 1,2,3,\ldots, from\ ground\ singlet\ state\ to\ excited\ n^{th}\ singlet\ state$). For a given transition (i.e., $n = 1,2,3,\ldots$), k_{ox} is independent of the photon energy, hv, because regardless of which vibronic state is excited by the photon energy, C_{60}** first relaxes vibrationally to the lowest energy vibronic state (Kasha's principle). Also, C_{60}* and $^1O_2$ after photosensitization are always the same C_{60}* and $^1O_2$ (indistinguishable of the excitation history), respectively, regardless of the photon energy for that particular optical excitation transition of C_{60} (i.e., $S_o \longrightarrow S_n,\ n = 1,2,3,\ldots$). Therefore, per a given optical excitation (transition), k_{ox} as well as Φ_e have no spectral dependence. In conclusion, $\sigma_e(v)$ and $\sigma_s(v)$ have the same line shape per a given optical excitation (e.g., $1\ ^1A_g \longrightarrow 2\ ^1H_u$). On the other hand the overall excitation spectra for emission and sensitization (superposition of peaks associated with multiple transitions) may be different, because the ratio of cross sections ($\sigma_e : \sigma_s$) may change from transition to transition (peak to peak) due to variation of k_{ox} from transition to transition (peak to peak).

Similarly, for oxidation, $\sigma_{ox}(v) = \sigma_e(v)[k_{ox}/k_e]$ from the above analysis. Hence, $\sigma_{ox}(v)$ and $\sigma_e(v)$ have the same line shape for a given excitation transition (i.e., $S_o \longrightarrow S_n,\ n = 1,2,3,\ldots$). Thus, excitation spectra for $^1O_2$ phosphorescence and C_{60} photooxidation should have the same line shape, if they are both excited through $1\ ^1A_g \longrightarrow 2\ ^1H_u$.

Computation of the quantum yield for photooxidation through $1^1A_g \rightarrow 2^1H_u$

In Figure 5a, excitation spectrum for $^1O_2$ phosphorescence (at 1270 nm, generated by excitation of C_{60}) is shown in red, and the absorption spectrum of C_{60} is shown in black. As established in Supplementary Reference 7, the quantum yield for photosensitization of $^1O_2$ by C_{60} (Φ_s) is close to unity in the visible range. Therefore, in Figure 5a, we overlap the red and black curves at the visible range (excitation spectrum for $^1O_2$ phosphorescence and absorption spectrum of C_{60}), so the ratio of the height of the red curve to that black curve at any wavelength equals Φ_s. As such, Φ_s drops to 10% at 299 nm, being the peak of the 2^1H_u band, as shown in Figure 5c. This is the overall Φ_s at 299 nm, which is total number of $^1O_2$ sensitized, divided by total number of photons absorbed. Additionally, as reported by Supplementary Reference 17, the optical absorption of C_{60} at 299 nm is essentially contributed by the 2^1H_u alone. Therefore, the quantum yield of $^1O_2$ sensitization by 2^1H_u is computed as 10%.

However, this number (i.e., 10%) is not the quantum yield for photooxidation. The sensitized $^1O_2$ does not undergo reaction with C_{60}* at 100% efficiency. The probability of the oxidation reaction for the $^1C_{60}$*•$^1O_2$ complex is $\frac{k_{ox}}{k_q+k_r+k_{ox}}$, where k's are the rate constants for oxidation, $^1O_2$ quenching and $^1C_{60}$* relaxation. Since k_q is solvent dependent, so is this probability, being computed as 0.053 for CCl_4. Hence, as questioned by the Reviewer, finally we can compute the quantum yield for oxidation of C_{60} after by excitation through 2^1H_u state as: $10\% \times 0.053 = 0.53\%$.

Supplementary References

1. Taylor, R. *et al.* Degradation of C_{60} by light. **351**, 277–277 (1991).

2. Creegan, K. M. *et al.* Synthesis and characterization of $C_{60}O$, the first fullerene epoxide. *J. Am. Chem. Soc.* **114**, 1103–1105 (1992).

3. Chibante, L. P. F. & Heymann, D. On the geochemistry of fullerenes: Stability of C_{60} in ambient air and the role of ozone. *Geochim. Cosmochim. Acta* **57**, 1879–1881 (1993).

4. Heymann, D. *et al.* $C_{60}O_3$, a fullerene ozonide: Synthesis and dissociation to $C_{60}O$ and O_2. *J. Am. Chem. Soc.* **122**, 11473–11479 (2000).

5. Murdianti, B. S. *et al.* C_{60} oxide as a key component of aqueous C_{60} colloidal suspensions. *Environ. Sci. Technol.* **46**, 7446–7453 (2012).

6. Fortner, J. D. *et al.* Reaction of water-stable C_{60} aggregates with ozone. *Environ. Sci. Technol.* **41**, 7497–7502 (2007).

7. Arbogast, J. W. *et al.* Photophysical properties of C_{60}. *J. Phys. Chem.* **95**, 11–12 (1991).

8. Wood, J. M. *et al.* Oxygen and methylene adducts of C_{60} and C_{70}. *J. Am. Chem. Soc.* **113**, 5907–5908 (1991).

9. Taliani, C. *et al.* Light-induced oxygen incision of C_{60}. *J. Chem. Soc., Chem. Commun.* 220–222 (1993).

10. Juha, L., Hamplová, V., Kodymová, J. & Špalek, O. Reactivity of fullerenes with chemically generated singlet oxygen. *J. Chem. Soc., Chem. Commun.* 2437–2438 (1994).

11. Juha, L. *et al.* The Role of the Oxygen Molecule in the Photolysis of Fullerenes. in *Fullerene Science and Technology* **8**, 289–318 (2000).

12. Schuster, D. I., Baran, P. S., Hatch, R. K., Khan, A. U. & Wilson, S. R. The role of singlet oxygen in the photochemical formation of $C_{60}O$. *Chem. Commun.* 2493–2494 (1998).

13. Juha, L. *et al.* Fast degradation of fullerenes by ultraviolet laser radiation. *Appl. Phys. B* **B57**, 83–84 (1993).

14. *Solubility Data Series*. *International Union of Pure and Applied Chemistry* **7**, (Pergamon Press, 1981).

15. Sato, T., Hamada, Y., Sumikawa, M., Araki, S. & Yamamoto, H. Solubility of oxygen in organic solvents and calculation of the Hansen solubility parameters of oxygen. *Ind. Eng. Chem. Res.* **53**, 19331–19337 (2014).

16. Shirono, K., Morimatsu, T. & Takemura, F. Gas solubilities (CO_2, O_2, Ar, N_2, H_2, and He) in liquid chlorinated methanes. *J. Chem. Eng. Data* **53**, 1867–1871 (2008).

17. Menéndez-Proupin, E., Delgado, A., Montero-Alejo, A. L. & García de la Vega, J. M. The absorption spectrum of C_{60} in n-hexane solution revisited: Fitted experiment and TDDFT/PCM calculations. *Chem. Phys. Lett.* **593**, 72–76 (2014).

18. Foote, C. S. Photophysical and Photochemical Properties of Fullerenes. in *Electron Transfer I. Topics in Current Chemistry* (ed. Mattay, J.) **169**, 348–363 (Springer-Verlag, 1994).

19. Whang, A. J., Chen, Y. & Wu, B. Innovative design of cassegrain solar concentrator system for indoor illumination utilizing chromatic aberration to filter out ultraviolet and infrared in sunlight. *Sol. Energy* **83**, 1115–1122 (2009).

20. Gates, M. D. Spectral distribution of solar radiation at the earth's surface. *Science* **151**, 523–529 (1966).

21. Schmidt, R. Influence of heavy atoms on the deactivation of singlet oxygen ($^1\Delta_g$) in solution. *J. Am. Chem. Soc.* **111**, 6983–6987 (1989).
